# Genetic landscape of autism spectrum disorder in Vietnamese children

**DOI:** 10.1038/s41598-020-61695-8

**Published:** 2020-03-19

**Authors:** Kien Trung Tran, Vinh Sy Le, Hoa Thi Phuong Bui, Duong Huy Do, Ha Thi Thanh Ly, Hieu Thi Nguyen, Lan Thi Mai Dao, Thanh Hong Nguyen, Duc Minh Vu, Lien Thi Ha, Huong Thi Thanh Le, Arijit Mukhopadhyay, Liem Thanh Nguyen

**Affiliations:** 1 Vinmec Research Institute of Stem Cell and Gene Technology, 458 Minh Khai, Hai Ba Trung district, Hanoi, Vietnam; 2 University of Engineering and Technology, Vietnam National University Hanoi, 144 Xuan Thuy, Cau Giay ditrict, Hanoi, Vietnam; 3 Department of Gene Technology, Hi-tech Center, Vinmec International Hospital, 458 Minh Khai, Hai Ba Trung district, Hanoi, Hanoi Vietnam; 4 Translational Medicine Laboratory, Biomedical Research Centre, School of Science, Engineering and Environment, University of Salford, Manchester, M5 4WT United Kingdom

**Keywords:** Molecular biology, Genetic predisposition to disease

## Abstract

Autism spectrum disorder (ASD) is a complex disorder with an unclear aetiology and an estimated global prevalence of 1%. However, studies of ASD in the Vietnamese population are limited. Here, we first conducted whole exome sequencing (WES) of 100 children with ASD and their unaffected parents. Our stringent analysis pipeline was able to detect 18 unique variants (8 *de novo* and 10 ×-linked, all validated), including 12 newly discovered variants. Interestingly, a notable number of X-linked variants were detected (56%), and all of them were found in affected males but not in affected females. We uncovered 17 genes from our ASD cohort in which *CHD8*, *DYRK1A*, *GRIN2B*, *SCN2A*, *OFD1* and *MDB5* have been previously identified as ASD risk genes, suggesting the universal aetiology of ASD for these genes. In addition, we identified six genes that have not been previously reported in any autism database: *CHM*, *ENPP1*, *IGF1*, *LAS1L, SYP* and *TBX22*. Gene ontology and phenotype-genotype analysis suggested that variants in *IGF1*, *SYP* and *LAS1L* could plausibly confer risk for ASD. Taken together, this study adds to the genetic heterogeneity of ASD and is the first report elucidating the genetic landscape of ASD in Vietnamese children.

## Introduction

Autism spectrum disorder (ASD) is a group of neurodevelopmental disorders that includes autism, Asperger’s syndrome and pervasive developmental disorder not otherwise specified and is characterized by restricted repetitive behaviour, delays in language development and lack of reciprocal social communication and interaction^1^. The common psychiatric and cognitive comorbidities with ASD include intellectual disability (ID)^2–4^, sleep deprivation^5,6^ and epilepsy (EP)^3,7^.

Recent studies have gradually emphasized the role of genetics in ASD, with up to 25% of ASD cases having genetically identifiable causes^8^. Males have a 4-fold higher incidence of ASD than females^9^, suggesting the involvement of genetics in the aetiology of ASD. Previous studies have shown that 5% to 15% of patients with ASD have inherited copy number variations (CNVs) or *de novo* CNVs in some affected genes with synaptic function^10^. Genome-wide association studies have indicated that common variations are risk factors for ASD^11,12^. Meanwhile, *de novo* CNV and loss of function (LoF) mutations merely explain 2.6% of the variance in liability^12^. Other studies have revealed that non-coding *de novo* mutations in the promoter regions also affect ASD^13,14^. Recent genomic studies with large samples have explored new genes linked to ASD^15,16^. To date, over 100 genes have been identified as strongly linked to the risk of ASD^17,18^ and enriched in the following three main pathways: chromatin remodelling, transcription and splicing, and synaptic function^18–20^. In fact, ASD is highly genetically heterogeneous due to multiple familial inheritance patterns and the occurrence of a large number of *de novo* variations. It is estimated that up to 1000 genes are potentially implicated ASD^21,22^, making it one of the most complex disorders. Genetic factors are known to play an important role in ASD aetiology, but that their elucidation is a work in progress^23–25^.

The prevalence of ASD has been recently reported as high as 16.8 per 1000 or 1/59 children in the US^9^. Unlike that in Western populations, ASD is not well studied in Asia, where the prevalence of ASD varies from studies and populations, ranging from 0.09–1.07% in south Asia^26^, 0.1018% in China^27^ and 2.64% in Korea^4^. Another study estimated that the prevalence of ASD in Asia from 1980 to recent present is 14.8/10,000 children^28^. A comprehensive study on 17,277 children aged 18 to 30 months in Vietnam revealed that the prevalence was 0.752%, and the number continues to increase^29^. It is estimated that ASD affects from 1% to 2% of the global population^4,30,31^. The reason for the varied prevalence of ASD across populations remains unclear, although sociodemographic factors such as socioeconomic status, ethnicity and parental education level have been reported to influence the diagnosis of ASD, resulting in disparities in ASD prevalence across populations^32–36^. With an increasing number of new variants/genes associated with ASD and different prevalence rates of ASD, we wanted to help shed light on whether genetic variation linked to ASD varies between populations. Thus, we conducted whole exome sequencing (WES) using a trio-based approach to investigate the genetic pattern of Vietnamese children with ASD.

## Results

### Clinical characteristics

We initially recruited 105 children who were definitively diagnosed with ASD and their unaffected parents. However, five families dropped out during recruitment. Finally, 100 trios were selected to participate in this study. In general, observations across populations, including Vietnamese populations, indicate that the ASD-affected male/female (M/F) ratio is approximately 4-fold^29,37,38^. The ratio of M/F with ASD in our study was slightly higher than this value (M/F: 4.9-fold). By the enrolment time, the proband’s age ranged from 3 to 17 years old (y/o). The average age was 6.9 y/o (Supplementary Table S1). The CARS scores ranged from 35 to 55.5, with an average score of 46.8 points. The DSM-V results showed that most probands were ranked at level 3 or required very substantial support, accounting for 68% of the cases. Denver II, comprising five domains including “Personal-social”, “Fine motor”, “Gross motor”, “Understanding” and “Language”, indicated that all probands had a lower developmental level compared to their chronological age. Overall, our cohort included ASD patients from mild to severe levels (Supplementary Table S1).

In addition to common autistic symptoms such as repetitive behaviours, speech delay, jargon, hyperactivity, toe walking and spinning, a quarter of the probands exhibited comorbid conditions such as ID (13 probands), EP (two probands), seizure (seven probands), ADHD (three probands) and cerebral palsy (CP) (one proband). Two cases showed congenital foot defects (ASD025 and ASD082) (Supplementary Table S1). Notably, six subjects had siblings with ASD (ASD004, ASD027) or with other neurological conditions, such as speech delay, CP and ID (ASD062, ASD065, ASD072 and ASD075). Of these, proband ASD004 and his male sibling were twins conceived through *in vitro* fertilization (IVF), while proband ASD027 and his sibling were monozygotic twins (Supplementary Table S1). Twenty-three probands showed language regression during their early life. Six probands often experienced gastrointestinal (GI) problems, mostly chronic constipation and diarrhoea. We were able to perform brain fluorodeoxyglucose (FDG)-PET/CT indicating hypometabolism of 29 probands and brain MRI of 40 cases (Supplementary Table S1). Brain MRI in 15 probands showed abnormal signal changes, while the rest showed either no abnormality detected or unavailable information.

### Screening of Rett and Fragile-X syndrome

Rett syndrome (MIM#312750) is a severe neurodevelopmental disorder that almost exclusively affects females. The disease is caused by heterozygous mutations in the X-linked *MECP2* gene, which encodes methyl-CpG binding protein 2. We screened all 17 ASD females for mutations in exons 2, 3 and 4 of the *MECP2* gene but did not find any potential causal variants. In other aspect, Fragile-X syndrome (MIM#30064) is caused by an expansion repeat of CGG in the 5′ UTR of the *FMR1* gene. This syndrome is a common comorbid condition with ASD as well as ID^39–41^. Therefore, we next screened for this syndrome in all 100 children with ASD. Surprisingly, no subjects in our study had changes in the *FMR1* gene. The patients with no mutations found in these two screening tests and their parents were then selected for the WES experiment.

### Variant identification

**Figure 1:**
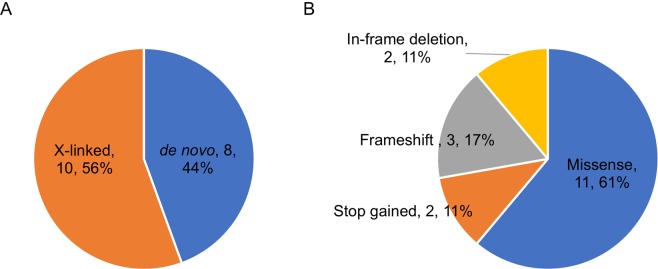
Types of validated variants and inheritance modes. (**A**) The distribution of inheritance modes; (**B**) The distribution of variant types. The values indicate the number of variants, and their percentages are presented in parentheses.

Paired-end sequencing resulted in 36.4 billion reads from 100 trios with an average Q30 score of 95.2. The average sequencing depth in the target region of all samples was 83×, ranging from 52 to 146.7× (Supplementary Table S2). All samples passed the quality control recommended by the manufacturer (Illumina). Although all the parents had self-claimed that they were biological parents to their children, our relatedness analysis revealed unclear parental relations in four cases (ASD080, ASD091, ASD100 and ASD101). In addition, the mother’s sample from case ASD034 was inadvertently sequenced twice (Supplementary Table S3). Therefore, we excluded these cases from the downstream analyses. Reads with low depth, variants with MAF > 0.1%, synonymous variants and variants predicted as benign, tolerated or neutral by PolyPhen-2, SIFT and MutationTaster were removed accordingly. We finally detected and Sanger validated a total of 18 unique variants in 17 genes from 16 unrelated probands. X-linked variants predominated, accounting for 56% of the total, followed by *de novo* variants (44%) (Fig. 1a, Table 1, Supplementary Fig. S1).

**Table 1:** Detected variants in 100 Vietnamese children with ASD.

Proband/Gender	Chr	Position	Gene	Variant			Genotype			gnomAD	MAF (%)	Inheritance
				SNP ID	Base change	Amino acid change	Proband	UM	UF	pLI/Z score	gnomAD/gnomAD East Asia/1000 G	
ASD005/M	X	79279657	*TBX22*	rs368136178	c.452 G > T	p.Arg151Leu	T	G/T	G	0.98/−0.13	0.007811/0.04724/0.0529801	X-linked
ASD006/F	12	102813438	*IGF1*	.	c.251 G > A	p.Arg84Gln	C/T	C/C	C/C	0.27/1.54	n/a	*De novo*
ASD032/M	2	149216339	*MBD5*	.	c.14_15delAA	p.Lys5fs	CAA/C	CAA/CAA	CAA/CAA	1/1.14	n/a	*De novo*
ASD035/M	X	76938776	*ATRX*	.	c.1972C > T	p.Arg658Cys	A	G/A	G	1/3.1	0.0005476/0.007216	X-linked
ASD038/M	21	38858853	*DYRK1A*	.	c.601 C > T	p.Gln201*	C/T	C/C	C/C	1/3.34	n/a	*De novo*
ASD046/M	12	13722914	*GRIN2B*	.	c.2208dupG	p.Asn737fs	T/TC	T/T	T/T	1/5.42	n/a	*De novo*
	X	115304290	*AGTR2*	.	c.757 C > T	p.Gln253*	T	C/T	C	0.01/−0.07	n/a	X-linked
ASD056/M	6	132185700	*ENPP1*	.	c.1081_1083delAAA	p.Lys361del	TAA/T	TAAA/TAAA	TAAA/TAAA	0/1.62	n/a	*De novo*
ASD057/M	X	32663092	*DMD*	.	c.1138 C > T	p.His380Tyr	A	G/A	G	1/−2.43	n/a	X-linked
ASD059/M	X	32490353	*DMD*	.	c.2877 A > C	p.Glu959Asp	G	T/T	T		n/a	X-linked
ASD063/M	14	21871315	*CHD8*	.	c.3575 T > C	p.Ile1192Thr	A/G	A/A	A/A	1/5.95	n/a	*De novo*
ASD068/F	2	166152561	*SCN2A*	rs34411329	c.232delC	p.Leu78fs	GC/G	GC/GC	GC/GC	1/6.46	n/a	*De novo*
ASD076/M	X	49050795	*SYP*	rs782025908	c.251 C > G	p.Ala84Gly	C	G/C	G	0.92/1.33	0.0005482/0.007216	X-linked
	X	64737988	*LAS1L*	.	c.1797_1805delTGATGAAGA	p.Asp599_Glu601del	C	CTCTTCATCA/C	CTCTTCATCA	1/2.67	0.03504/0.05409	X-linked
ASD083/M	X	13778788	*OFD1*	rs778936071	c.2209 A > G	p.Thr737Ala	G	A/G	A	0.96/0.32	0.005428/0.07444	X-linked
ASD086/M	X	85212934	*CHM*	.	c.866 T > C	p.Met289Thr	G	A/G	A	1/0.79	0.001099/0	X-linked
ASD097/M	12	60165042	*SLC16A7*	rs141923225	c.260 C > T	p.Pro87Leu	C/T	C/C	C/C	0.01/0.28	0.001598/0	*De novo*
ASD099/M	X	153693430	*PLXNA3*	rs201083788	c.2113 C > T	p.Arg705Trp	T	C/T	C	0.05/0.68	0.004982/0.06801/0.0529801	X-linked

Chr (Chromosome); MAF represents the minor allele frequency that appeared in gnomAD and 1000 Human Genome Project; n/a (not available); UF (unaffected father); UM (unaffected mother).

We observed that only six variants were recorded in dbSNP (Build 152), and the remaining variants were newly uncovered (Table 1). Missense variants were the major events, accounting for 61% of the total detected variants, followed by frameshift (17%), in-frame deletion (11%) and stop gained variants (11%) (Fig. 1b). Interestingly, we found two different variants in the *DMD* gene from two unrelated probands (ASD057 and ASD059) (Tables 1, 2). We used SnpEff, SIFT, PolyPhen-2, MutationTaster and CADD to predict the deleteriousness of the nucleotide changes. SnpEff predicted that five different variants detected from four individuals had a high impact (Table 2), and all of them were LoF variants (MDB5:c.14_15delAA, DYRK1A:c.601 C > T, GRIN2B:c.2208dupG, AGTR2:c.757 C > T and SCN2A:c.232delC), while the rest had a moderate impact. Two variants, DYRK1A: c.601 C > T (proband ASD038) and AGTR2:c.757 C > T (proband ASD046), had the highest CADD scores, suggesting these variants were most deleterious (Table 2). Two missense variants, CHM:c.866 T > C (proband ASD086) and SLC16A7:c.260 C > T (proband ASD097), were predicted to be benign by PolyPhen-2 but were damaging by MutationTaster and SIFT. The remaining variants were predicted to be damaging or disease causing by all employed *in silico* tools (Table 2).

**Table 2:** *In silico* predictions of identified variants.

Proband (Gender)	Gene	Variant		*In silico* prediction				Impact	Type of variant
		Base change	Amino acid change	CADD score (scaled)	PolyPhen-2 (score)	Mutation Taster (score)	SIFT (score)		
ASD005/M	*TBX22*	c.452 G > T	p.Arg151Leu	24.9	P(0.703)	D(1)	D(0.001)	Moderate	Missense
ASD006/F	*IGF1*	c.251 G > A	p.Arg84Gln	27.2	D(0.998)	D(1)	D(0.001)	Moderate	Missense
ASD032/M	*MBD5*	c.14_15delAA	p.Lys5fs			D(1)		High	Frameshift
ASD035/M	*ATRX*	c.1972C > T	p.Arg658Cys	24.7	D(0.993)	D(1)	D(0)	Moderate	Missense
ASD038/M	*DYRK1A*	c.601 C > T	p.Gln201*	37		A(1)		High	Stop gained
ASD046/M	*GRIN2B*	c.2208dupG	p.Asn737fs					High	Frameshift
	*AGTR2*	c.757 C > T	p.Gln253*	36		D(1)		High	Stop gained
ASD056/M	*ENPP1*	c.1081_1083delAAA	p.Lys361del			D(0.547)		Moderate	In-frame deletion
ASD057/M	*DMD*	c.1138 C > T	p.His380Tyr	24.5	D(0.994)	D(0.999)	D(0.015)	Moderate	Missense
ASD059/M	*DMD*	c.2877 A > C	p.Glu959Asp	24.5		D(0.0999)		Moderate	Missense
ASD063/M	*CHD8*	c.3575 T > C	p.Ile1192Thr	27.1	D(0.948)	D(1)	D(0)	Moderate	Missense
ASD068/F	*SCN2A*	c.232delC	p.Leu78fs			D(1)		High	Frameshift
ASD076/M	*SYP*	c.251 C > G	p.Ala84Gly	25.3	P(0.816)	D(1)	D(0.002)	Moderate	Missense
	*LAS1L*	c.1797_1805delTGATGAAGA	p.Asp599_Glu601del					Moderate	In-frame deletion
ASD083/M	*OFD1*	c.2209 A > G	p.Thr737Ala	24	D(0.997)	D(0.943)	D(0.005)	Moderate	Missense
ASD086/M	*CHM*	c.866 T > C	p.Met289Thr	24.5	B(0.322)	D(1)	D(0.013)	Moderate	Missense
ASD097/M	*SLC16A7*	c.260 C > T	p.Pro87Leu	24.5	B(0.427)	D(1)	D(0.035)	Moderate	Missense
ASD099/M	*PLXNA3*	c.2113 C > T	p.Arg705Trp	27.2	P(0.772)	D(1)	D(0)	Moderate	Missense

Impact of variants predicted by PholyPhen-2, SIFT, MutationTaster and CADD where the symbol and the score in the parentheses indicate the impact. PolyPhen-2 includes three categories (B: benign; P: possibly damaging; D: damaging; score value close to 1 indicates likely damaging/deleterious); SIFT (D: damaging; T: tolerated; score value <0.05 is likely damaging/deleterious); MutationTaster (A: disease causing automatic; D: disease causing; N: polymorphism; P: polymorphism automatic; score value close to 1 shows a high security of the prediction); CADD (scaled score; the higher the value, the more deleterious the mutation is).

### Phenotype-genotype analysis and biological function

In total, we obtained 17 different genes, which showed genetic predispositions from 16 unrelated probands. We used the three most popular autism databases, SFARI (https://gene.sfari.org/), AutDB^42^ and the syndromic category in AutismKB^43^, to investigate the association of the detected genes with ASD. We found that 11/17 genes were previously reported in SFARI and AutDB (Table 3). Meanwhile, only four genes, *AGTR2*, *ATRX*, *DMD* and *MBD5*, were categorized as syndromic ASD genes in AutismKB. We next compared our genes to gene sets of ASD or other neurological disorders and found that *CHD8*, *DYRK1A*, *GRIN2B*, *MBD5* and *SCN2A* overlapped with the ASD gene sets (FDR ≤ 0.1) reported in previous large-scale ASD studies^44,45^. *CHD8*, *DYRK1A*, *GRIN2B* and *SCN2A* were found in a gene set containing 94 genes enriched in developmental disorders derived from the DECIPHER project^46^. Three genes (*DYRK1A*, *GRIN2B* and *SCN2A*) were found in the EP gene set^47^. Hence, these genes detected in our ASD cohort are highly implicated in ASD or other neurological disorders.

**Table 3:** ASD-associated genes, biological function and phenotype analysis.

GENE	HGNC	Encoding protein; function	Biological process; Pathway	SFARI (score)	AutDB	AutismKB (syndromic)	HPO	Linked to ASD or other neurological disorders
*AGTR2*	338	Angiotensin II receptor type 2	Blood circulation; Cellular response to peptide hormone stimulus; Inflammatory response; Regulation of anatomical structure size	+ (4)	+	+	n/a	X-linked MR 88, EP
*ATRX*	886	Transcriptional regulator ATRX; Involvement in the gene regulation at interphase and chromosomal segregation in mitosis	n/a	+ (4)	+	+	Alpha-thalassemia, MR syndrome, X-linked, Myelodysplasia, Neuroendocrine tumour of stomach	ASD, DD, EP, ID
*CHD8*	20153	Chromodomain helicase DNA binding protein 8; Transcriptional regulation, epigenetic remodelling, promotion of cell proliferation, and regulation of RNA synthesis	Wnt signalling	+ (1 S)	+		ASD	ASD, DD, ID, ADHD, SCZ
*CHM*	1940	Rab proteins geranylgeranyltransferase component A1; Involved in geranylgeranyl transfer reaction	Intracellular protein transport; Intracellular signal transduction; Nervous system process; Neurotransmitter secretion; Vesicle-mediated transport				Choroideremia	
*DMD*	2928	Dystrophin; Bridging the inner cytoskeleton and the extracellular matrix	Muscle fibre and organ development, positive regulation of neuron differentiation, neuron projection development, extracellular matrix	+ (S)	+		Becker/ Duchene muscular dystrophy, cardiomyopathy dilated X-linked type 3B	ASD, ADHD, DD, EP, ID, SCZ
*DYRK1A*	3091	Dual specificity tyrosine-phosphorylation-regulated kinase 1A; Cell proliferation, brain development.	Cell cycles, miotic G1-G1/S phases	+ (1 S)	+		MR autosomal dominant type 7	ASD, DD, EP, ID
*ENPP1*	3356	Ectonucleotide pyrophosphatase/phosphodiesterase 1	ATP metabolic process; Biomineral tissue development; Cell differentiation; Inorganic anion transport; Nucleoside triphosphate catabolic process; Ossification				Hypophosphatemic rickets; Arterial calcification; Diabetes; Pseudoxanthoma Elasticum; Obesity; Cole disease	
*GRIN2B*	4586	Glutamate ionotropic receptor NMDA type subunit 2B; Brain development, circuit formation, synaptic plasticity, cellular migration and differentiation.		+ (1)	+		MR; West syndrome; Epileptic encephalopathy	ASD, ADHD, DD, EP, ID, SCZ.
*IGF1*	5464	Insulin-like growth factor I; Involved in mediating growth and development.	Insulin/IGF pathway				Insulin-like growth factor I deficiency	
*LAS1L*	25726	LAS1 like, ribosome biogenesis factor	Maturation of 5.8 S rRNA; Maturation of LSU-rRNA				Wilson-Turner X-linked MR syndrome	
*MBD5*	20444	Methyl-CpG binding domain protein 5; Involved in cell division, growth and differentiation.	Post-translational protein modification; Metabolism of proteins	+ (4)	+	+	2q23.1 microdeletion syndrome	ASD; ADHD, DD, EP, ID, SCZ
*OFD1*	2567	OFD1, centriole and centriolar satellite protein	Cell cycle; Centrosome maturation; Signalling by Hedgehog; Organelle biogenesis and maintenance	+ (4)	+		Primary ciliary dyskinesia; Oral-facial-digital syndrome type 1; Simpson-Golabi-Behmel syndrome type 2; Joubert syndrome type 10	ASD
*PLXNA3*	9101	Plexin A3	Axon guidance; Cell adhesion and migration; Cell surface receptor signalling pathway; Regulation of GTPase activity	+ (4)	+		n/a	ASD
*SCN2A*	10588	Sodium voltage-gated channel alpha subunit 2; Involved in the generation and propagation of action potentials in neurons and muscle	Action potential; Chemical synaptic transmission; Nervous system process	+ (1)	+		Seizures; Benign familial neonatal infantile; Epileptic encephalopathy;Dravet syndrome; West syndrome	ASD, ADHD, DD, EP, ID
*SLC16A7*	10928	Solute carrier family 16 member 7	Transport of bile salts and organic acids, metal ions and amine compounds	+ (5)	+		n/a	ASD
*SYP*	11506	Synaptophysin	Synaptic vesicle trafficking				X-linked non-syndromic ID;	
*TBX22*	11600	T-box transcription factor; Involved in the regulation of developmental processes	Regulation of transcription by RNA polymerase II; Transcription by RNA polymerase II				Cleft palate; X-linked; Charge-like syndrome; Abruzzo-Erickson syndrome	

Reported genes in the database are indicated as “+” symbol; Scores of the ASD genes in SFARI are presented in the parentheses: Syndromic ASD (S); High confidence, syndromic (1 S); High confidence (1); Strong candidate (2) Suggestive evidence (3); Minimal evidence (4); Hypothesized (5); Not supported (6); and “no rating”. ASD (Autism spectrum disorder); ADHD (Attention-deficit/hyperactivity disorder); BPD (Bipolar disorder); DD (Developmental delay); EP (Epilepsy); ID (Intellectual disability); MR (Mental retardation); SCZ (Schizophrenia); n/a (not available); HPO (Human phenotype ontology).

Interestingly, our data showed six ASD candidate genes, *CHM*, *ENPP1*, *IGF1*, *LAS1L*, *SYP* and *TBX22*, which have not been previously reported in the three aforementioned autism databases (Table 3). Human Phenotype Oncology (HPO) analysis showed that all of these candidate genes have been reported to be linked to human diseases (Table 3). Mutations in the *IGF1*^48,49^, *LAS1L*^50,51^ and *SYP*^52^ genes have been found in patients with CP and ID. In our study, a *de novo* missense variant was detected in the *IGF1* gene from a female proband (ASD006) whose language and intellectual status were regressed after 18 m/o. Her language and personal-social status were strongly delayed (Supplementary Table S1). Brain PET-CT showed a decrease in FDG uptake in the temporal lobe, bilateral hippocampus, prefrontal region and parietal lobe (Supplementary Tables S1,S4). *IGF1* mutations have been previously found in individuals with hyperactivity and short intension^48,49^. Treatment with *IGF1* or expression of *SHANK3*, which is associated with idiopathic ASD, may restore synaptic deficits in neurons from Phelan-McDermid syndrome^53,54^. We thus conferred that IGF1 deficiency may reduce the synaptic transmission of neurons.

**Figure 2:**
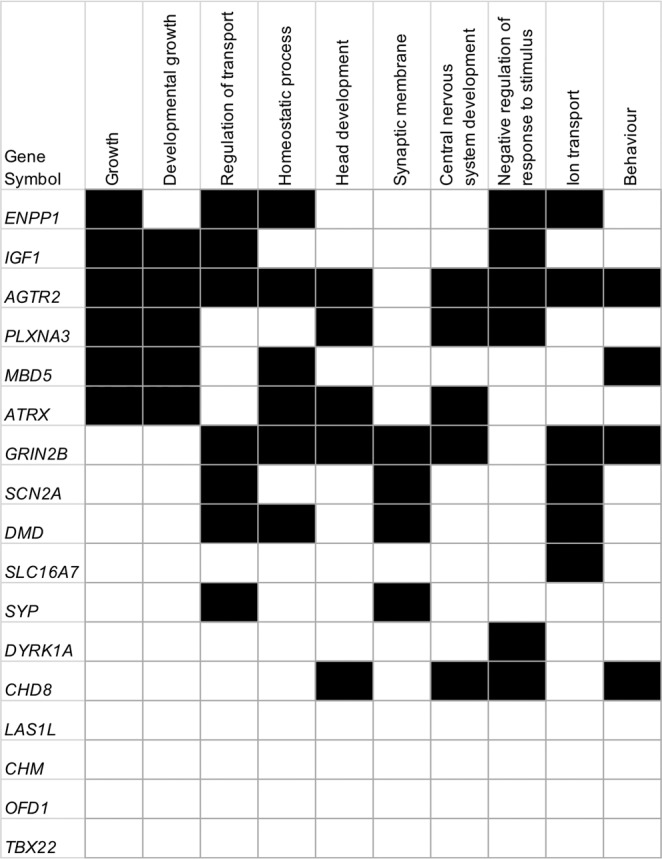
Gene ontology analysis. Candidate genes and the gene sets are listed in the vertical line and in the upper horizontal line, respectively.

We also found two X-linked variants in the *LAS1L* and *SYP* genes from a male proband (ASD076). *SYP* encodes synaptophysin, which is involved in the regulation of synaptic plasticity^55^. GO analysis also showed that this gene is involved in synaptic membrane activity (Fig. 2, Supplementary Table S5). Moreover, several *SYP* mutations with evidence of segregation have been reported in patients with X-linked nonsyndromic mental retardation^52^. Therefore, *SYP* is a plausible gene for ASD. In addition, *LAS1L* encodes the ribosomal biogenesis protein LAS1L, which is required for cell proliferation, ribosome biosynthesis of the 60 S ribosomal subunit and the maturation of 28 S rRNA^56,57^. Missense mutations in the *LAS1L* gene have been detected in individuals with Wilson-Turner X-linked mental retardation syndrome^50^ and in a proband with congenital lethal motor neuron disease^51^. Our proband showed language regression after 12 m/o. At 5 y/o, he remained nonverbal and progressively developed more severe symptoms (Supplementary Table S4). Brain PET-CT images showed severe hypometabolism in the hippocampus region and in the frontal lobe (Supplementary Table S1). Interestingly, we observed that two probands (ASD006, ASD076) who carried *IGF1*, *SYP* and *LAS1L* variants showed language regression and brain hypometabolism (Supplementary Tables S1,S4).

Gene ontology (GO) analysis of the 17 detected genes against 9996 gene sets derived from the Molecular Signatures Database (MSigDB) with a false discovery rate (FDR) <0.05 revealed that *AGTR2, PLXNA3, ATRX, GRIN2B* and *CHD8* overlapped in the GO categories head development and central nervous system development (Fig. 2, Supplementary Table S5). Four genes, *GRIN2B, SCN2A, DMD* and *SYP*, were found in the GO category synaptic membrane. We also observed that *ENPP1, IGF1, AGTR2, PLXNA3, DYRK1A* and *CHD8* were involved in the GO category negative regulation of response to stimulus. These results suggested that these particular biological processes might be related to ASD or other neurological conditions.

## Discussion

The rapidly declining cost of next-generation sequencing in recent years has resulted in many genetic studies of neurodegenerative disorders, including ASD^58–60^. As a result, an increasing number of new genes and variants have been found to be enriched in ASD individuals, revealing the highly heterogeneous nature of ASD. In addition, cutting-edge molecular biology or modelling of ASD allows us to better define and understand the aetiology of ASD and biological pathways of associated genes^61^. In this study, we performed whole exome sequencing of 100 Vietnamese children with ASD and their unaffected parents together with analysis of biological processes to explore the genetic landscape of ASD in Vietnamese children.

Gender bias between males and females in ASD is generally acknowledged, where males have an approximately 4-fold higher incidence of ASD than females. Several hypotheses on male bias, such as the nonrecognition of females with ASD^62,63^, differences in brain structure and genetic load between females and males^64^ and female protective effect against autistic behaviour^65^, have been proposed. It is hypothesized that females carry a higher genetic load and are thus less vulnerable to ASD from genetic causes than males^66^. Meanwhile, *de novo* variations are strongly associated with ASD, suggesting that females have a greater resistance to *de novo* variations than males^67,68^. Our findings with a predominance of males over females (4.9-fold) are consistent with those from previous studies^65,69–71^. Moreover, the results of all X-linked variants detected in affected males further supported the recent hypothesis of the female protective effect against ASD^72^.

Regarding the number of *de novo* variants detected, a previous study using whole genome sequencing from the 1902 quartet family with ASD reported 67.1 *de novo* variants per child’s genome (61 SNV and 5.6 indel DNV per child)^13^. Another study using WES^73^ detected 0.9 *de novo* variants per child’s exome (869 SNVs and 27 indel *de novo* variants in a total cohort of 990 ASD samples). In our study, before variant filtering by stringent conditions, we detected 1.3 *de novo* variants per child’s exome, which was compatible with the results of previous studies. After filtering, the *de novo* variant rate in our study was 0.08%, including three missense and five LoF variants (three frameshift, one stop gained and one in-frame deletion) (Table 1).

We observed that 11 genes from our cohort have been reported in the autism databases (Table 3). Among these genes, *CHD8, DYRK1A*, *GRIN2B*, *SCN2A*, and *MDB5* were highly recognized as ASD risk genes with FDR <0.1, as previously reported^17,18,44,45,74,75^. In addition, *OFD1* is involved in the Wnt pathway and is highly suggested as an ASD risk gene^76^. Most of these genes also overlapped in the gene sets of developmental disorders^46^ or EP^47^. Thus, these genes were highly universal for ASD regardless of population.

Given that ASD is highly genetically heterogeneous, there are several key biological processes involved in the development of ASD, including neurogenesis, synaptic plasticity, synaptogenesis and neurite growth^8,20,77^. Among the six newly uncovered genes in this study, we observed that *IGF1* and *SYP* encode proteins involved in brain development and synaptic activities, respectively. In addition, a dozen ASD-linked genes are involved in several biological processes, such as ribosomal maturation and mRNA regulation, that are linked to synaptic function and chromosome condensation^18,20,78^. Defects in genes encoding ribosomal proteins have resulted in downregulation of these genes in children with ASD and healthy woman with autistic children^79^. Therefore, defects of *LAS1L*, which plays a role in ribosome biogenesis, likely lead to a disruption of ribosomal maturation.

We found 18 unique variants in 17 genes from 16 unrelated probands. Biological analysis showed that many of these genes were involved in neuronal activities and formation. Together with previously ASD-linked genes, other newly detected genes in this study, including *IGF1*, *LAS1L* and *SYP*, were found to be plausible ASD-related genes due to their involvement in some risk-associated biological processes and their associations with other neurological conditions. In general, ASD is a complex disorder showing a broad range of phenotypes, where 35% of ASD cases have ID, 5–15% have EP and 50% have language developmental delay^22^. Therefore, phenotype-genotype analysis accompanied by biological process/pathway analysis is critical for the determination of their associations with ASD. However, functional studies on these genes in ASD development should be performed.

## Conclusions

Consistent with previous studies, this study found a predominance of ASD-affected males over affected females (4.9-fold). Rett and Fragile-X syndrome are the most common comorbidities with ASD, but there was no individual in our cohort with these syndromes. With a stringent pipeline, we finally identified 18 unique variants that occurred in 17 genes, of which 12 variants were reported for the first time. All X-linked variants were detected in male probands but not in affected females. This finding is consistent with a contribution of X-linked recessive variants to ASD. We found 11 genes formally associated with ASD, some of which have been previously identified as ASD risk genes (*CHD8, DYRK1A*, *GRIN2B*, *SCN2A*, *OFD1* and *MDB5*), indicating the genetic universal aetiology of ASD. Interestingly, this study uncovered variants in six new candidate genes (*CHM, ENPP1, IGF1, LAS1L, SYP* and *TBX22*) enriched in our ASD cohort. Analyses of phenotype-genotype and GO showed that many of our detected genes were associated with several neurological conditions and were involved in some neuronal biological processes, such as synaptic processes, regulation of transport and ribosome maturation. We conferred that genetic predispositions in these genes might be causative factors. This study is the first to elucidate the common and unique genetic landscape of ASD in Vietnamese children.

## Methods

### Ethics statement

The study protocol was approved by the Ethical Committee of Vinmec International Hospital in accordance with the Declaration of Helsinki. Before the enrolments, written informed consent forms including the use of peripheral blood and clinical data for research use and publication were obtained from the parents.

### Study cohort and sample collection

Children with suspected ASD were independently examined by trained physicians and psychologists at Vinmec Times City International Hospital, Hanoi, Vietnam, using the Diagnostic and Statistical Manual of Mental Disorders, Fifth Edition (DSM-V)^1^, the Autism Diagnostic Observation Schedule (ADOS)^80^, and Childhood Autism Rating Scale (CARS)^81^. DSM-V classified the severity of ASD into three levels: Level 1 (requiring support), Level 2 (requiring substantial support) and Level 3 (requiring very substantial support). To examine the development of the probands, the Denver Developmental Screening test II^82^ (Denver II) was used. Development of the proband (in months) was examined based on five domains: personal-social, fine motor, gross motor, understanding and language. The developmental quotient (DQ) was calculated by dividing the average developmental age from five domains by the chronological age of the proband and multiplying by 100. Definitively diagnosed children with ASD and their unaffected parents were recruited to participate in the study. Approximately 3–4 mL of peripheral blood from children with ASD and their unaffected parents was collected and kept in EDTA tubes. Genomic DNA was extracted by using a QIAamp Blood Kit (Qiagen, Germany) and stored at −80 °C before use.

### Screening of Fragile-X and RETT syndrome

Fragile-X and Rett syndrome are the most common causes of inherited mental retardation and are reportedly linked to ASD. Therefore, this study aimed to exclude patients with either Fragile-X or Rett syndrome. An AmplideX FMR1 PCR kit (Asuragen, TX, USA) was used for screening Fragile-X syndrome. Genotypes were determined by examining the size of the trinucleotide repeat segment and the methylation status of the *FMR1* gene. Since Rett syndrome is predominantly found in females due to the mutations in the *MECP2* gene, all females diagnosed with ASD were selected for screening for mutations in exons 2, 3 and 4 of the *MECP2* gene^83^. A detailed method and primer list for *MECP2* mutation screening can be found in our previous study^84^. Direct sequencing was performed on an ABI 3500 DX system as indicated hereinafter.

### Whole exome sequencing

Probands without mutations detected in the *MECP2* and *FMR1* genes and their biological self-claimed parents were selected for the WES experiment. A DNA library was constructed by using a Nextera Rapid Capture Exome Kit (Illumina, USA) capturing over 98% of the exonic contents. The library concentration was quantified by a Qubit dsDNA Broad Range Assay Kit (Invitrogen, USA). Library size was measured by a Lab Chip 3 K Hisense Kit (Perkin Elmer, USA). Paired-end exome sequencing with a read length of 75 × 2 bp was performed on a HiSeq. 4000 (Illumina, USA).

### Data analysis

Adapters were removed prior to downstream analysis. BWA version 0.7.15 was used for alignment against the human reference genome version GRCh37^85^. Short, index and mark duplicates were assessed by using Samtool version 1.3^86^ and Picard version 2.7.2 (http://broadinstitute.github.io/picard/). GATK toolkit version 3.6^87^ and Platypus version 0.8.1^88^ were used to call variants (single nucleotide variants and indels with less than 50 bp). Variants were considered highly reliable if they were called by both GATK and Platypus with a Phred-score of equal or greater than 30. The minor allele frequency (MAF) was attained from the 1000 Human Genome Project^89^ and gnomAD database (https://gnomad.broadinstitute.org/). Biological relatedness was analysed by using Peddy software version v0.4.3^90^. Trio samples with non-biological relatedness were excluded from the downstream analyses.

### Variant classification

Variants were annotated by SnpEff programme version 4.3g^91^ and fulfilled the following criteria: (i) variant with MAF <0.1%^92^ against gnomAD, gnomAD East Asia, and 1000 Human Genomes Project; (ii) variant passed the GATK standard filters; (iii) nonsynonymous variants including missense variants with a prediction of damaging impact and LoF variants (nonsense, frameshift and splicing variants); and (iv) variant occurred in genes recorded in the DECIPHER and/or SFARI databases. Putative *de novo* variants were considered if independent reads in all family members were ≥20 and if both parents were homozygous for the reference and the offspring was heterozygous; an X-linked variant was considered if it occurred on the X chromosome.

### In silico prediction, gene function and biological process

PolyPhen-2 version 2.2^93^, SIFT version 4.0^94^ and MutationTaster^95^ were employed to predict the damaging impact of the missense variants. Combined Annotation-Dependent Depletion (CADD) GRCh37-v.14 was used to score the deleteriousness of variants with a single nucleotide change^96^. Gene function, protein class and biological process were determined by the PANTHER classification system v.14^97^. Validated gene sets were computed with the Molecular Signatures Database (MSigDB) v6.2^98,99^ to further explore the gene ontology of the ASD candidate genes. Human Phenotype Ontology (HPO-Web version 1.5.0)^100^ was used to find the association of candidate genes with human diseases.

### Variant validation

After the stringent filtering strategy, the identified variants were validated by Sanger sequencing. Proper primers were designed for each variant by using Primer3Plus software^101^. Fresh DNA was newly extracted from stored blood samples by using the same DNA extraction protocol. Target fragments were amplified by GoTaq DNA polymerase (Promega, USA). The amplicons were sequenced on an ABI 3500 DX system using a BigDye Terminator v3.1 (Thermo Fisher Scientific, USA). Both forward and reverse read alignment were used to determine the sequence.

## Supplementary information

Supplementary Information.

## Data Availability

Sequencing data generated and analysed in this study are included in this article and its Supplementary Information.
