# Supplementary material for: Genetic landscape of autism spectrum disorder in Vietnamese children
Source: Sci Rep. 2020 Mar 19;10:5034. doi: 10.1038/s41598-020-61695-8 (PMC7081304; doi:10.1038/s41598-020-61695-8)
Supplement: Supplementary file 1 — Supplementary Information. [file 41598_2020_61695_MOESM1_ESM.pdf]

Supplementary Information

Genetic landscape of autism spectrum disorder in Vietnamese children

Kien Trung Tran[1], Vinh Sy Le[1,2*], Hoa Thi Phuong Bui[3], Duong Huy Do[3], Ha Thi Thanh Ly[3], Hieu Thi Nguyen[1], Lan Thi Mai Dao[1], Thanh Hong Nguyen[1], Duc Minh Vu[3], Lien Thi Ha[3], Huong Thi Thanh Le[3], Arijit Mukhopadhyay[4], Liem Thanh Nguyen[1*]

[1]Vinmec Research Institute of Stem Cells and Gene Technology, 458 Minh Khai, Hai Ba Trung district, Hanoi, Vietnam.

[2]University of Engineering and Technology, Vietnam National University Hanoi, 144 Xuan Thuy, Cau Giay, Hanoi.

[3]Department of Gene Technology, Hi-tech Center, Vinmec International Hospital, 458 Minh Khai, Hai Ba Trung district, Hanoi, Vietnam.

[4]Translational Medicine Laboratory, Biomedical Research Centre, School of Science, Engineering and Environment, University of Salford, Manchester M5 4WT, United Kingdom.

Co-corresponding authors: Vinh Sy Le (vinhls@vnu.edu.vn); Liem Thanh Nguyen (v.liemnt@vinmec.com; Tel: +84439743556; Fax: +84439743557).

Supplementary Table S1. Clinical characteristics of patients with ASD

Proband/ Gender	DOB	Diagnosis: Age when diagnosed (month): First time	Diagnosis: Age when diagnosed (month): Enrolling	Diagnosis: DSM-V level	Diagnosis: ADOS score	Diagnosis: CARS score	Diagnosis: Denver II (month): PS/FM/GM/U/LA	Diagnosis: Denver II (month): DQ (%)	Clinical features: Delivery (weight at birth)/ Milestones & other presentations	Clinical features: Brain imaging: FDG-PET/CT (FDG hypometabolism)	Clinical features: Brain imaging: MRI
ASD002/M	2007	36	108	1-2	22/12	41	42/ 54/ 36/ 33/ 120	53	C-section (3.3 kg)/Walking at 12 m/o	n/a	NAD
ASD003/M	1999	30	209	3	21/12	50	39/ 36/ 209/ 23/ 9	30	Vaginal	n/a	n/a
ASD004/M	2010		89	3	20/12	50	29/ 29/ 54/ 26/ 16	34	C-section (2.8 kg)/IVF twins, sibling with ASD, walking at 14 m/o	Parietal lobe, central sulcus, hippocampus, anterior cingulate gyrus	n/a
ASD005/M	2013	36	45	3	24/12	50	14/ 18/ 21/ 8.5/ 12	33	C-section (3.8 kg), mother was 42 y/o when delivered/Walking at 24 m/o	n/a	n/a
ASD006/F	2011	18	78	2-3	21/12	47	24/ 51/ 48/ 39/ 27	48	C-section (3.4 kg)/Walking and babbling at 13 m/o, language and intellectual regression after 18 m/o	Temporal lobe, hippocampus, right parietal lobe	n/a
ASD008/F	2013	30	53	2	17/12	37	18/ 24/ 30/ 30/ 19	46	Vaginal (3.5 kg)/Walking at 16 m/o, constipation	n/a	n/a
ASD009/M	2010	30	88	3	23/12	51	27/ 24/ 48/ 21/ 13	30	C-section (3.5 kg)/ID	n/a	n/a
ASD010/M	2009	36	93	2	18/12	35	60/ 60/ 60/ 48/ 36	57	Vaginal (3.2 kg)/Walking and babbling at 12 m/o, language regression after 2.5 y/o, constipation	n/a	n/a
ASD011/M	2011	24	74	3	21/12	47	18/ 24/ 36/ 11/ 5	25	Vaginal (3.6 kg)/Babbling at 7-8 m/o, walking at 10 m/o, language regression after 18 m/o	Hippocampus, central sulcus, parietal lobe, anterior cingulate gyrus	n/a
ASD012/M	2003	18	161	2	20/12	43	66/ 54/ 162/ 48/ 27	44	C-section (3.2 kg)/Walking at 12 m/o, speech delay	n/a	n/a
ASD013/M	2012	24	60	3	21/12	51	19/ 25/ 36/ 17/ 15	37	C-section (3.2 kg)/Babbling at 10 m/o, walking at 13.5 m/o, language regression after 14-15 m/o	Temporal lobe, hippocampus, cerebellum	n/a
ASD014/M	2004	n/a	n/a	n/a	n/a	n/a	n/a	n/a	n/a	n/a	n/a
ASD015/M	2012	24	60	3	22/12	53	12/ 10/ 16/ 6.5/ 9	18	Vaginal (1.7 kg)/Preterm birth at 7th months of gestation, walking at age of 3, ID, DD, CP	n/a	ABN

Proband/ Gender	DOB	Diagnosis							Clinical features		
		Age when diagnosed (month)		DSM-V level	ADOS score	CARS score	Denver II (month)		Delivery (weight at birth)/ Milestones & other presentations	Brain imaging	
		First time	Enrolling				PS/FM/GM/U/LA	DQ (%)		FDG-PET/CT (FDG hypometabolism)	MRI
ASD016/M	2011	22	67	3	21/12	48	18/ 45/ 36/ 17/ 13	38	Preterm birth at 33 weeks of gestation (1.9 kg), GI problems	n/a	n/a
ASD017/F	2006	18	126	n/a	n/a	53	18/ 24/ 36/ 42/ 14	21	Vaginal (3.2 kg)/Walking at 12 m/o	n/a	ABN
ASD018/M	2003	18	160	3	21/12	49	39/ 48/ 48/ 23/ 18	22	Vaginal (3.8 kg)	n/a	n/a
ASD019/M	2012	24	49	2	20/12	37	21/ 45/ 27/ 42/ 30	67	C-section (3.6 kg)/Walking at 12 m/o, GI problems	n/a	n/a
ASD020/M	2013	18	38	3	21/12	46	19/ 39/ 36/ 20/ 14	67	Vaginal (3.7 kg)/walking and babbling at 13 m/o, ADHD	n/a	n/a
ASD021/M	2011	24	67	3	22/12	50	14/ 24/ 36/ 16/ 4.5	28	C-section (3.1 kg)/Walking at 22 m/o, ID	Anterior cingulate gyrus	n/a
ASD022/M	2011	18	72	3	20/12	55	17/ 23/ 36/ 14/ 6	27	C-section (3.2 kg)/Walking at 15 m/o, speech delay	Temporal lobe, bilateral hippocampus, prefrontal cortex	NAD
ASD023/M	2012	27	57	2-3	18/12	44	23/ 29/ 36/ 24/ 17	45	Vaginal (3.4 kg) at 41 weeks of gestation/Walking at 18 m/o	n/a	n/a
ASD024/F	2011	21	75	3	20/12	55	9.5/ 19/ 24/ 5.5/ 5.5	17	Vaginal preterm birth at 37 weeks of gestation (2.2 kg)/Walking at 14 m/o	Parietal lobe, central sulcus, temporal lobe, hippocampus, anterior cingulate gyrus	ABN
ASD025/F	2011	24	61	3	17/12	46	6/ 10/ 16/ 8.5/ 9	16	Vaginal (2.8 kg)/Flat feet, motor delay, seizure, ID	n/a	ABN
ASD026/M	2010	30	79	3	22/12	45	30/ 48/ 48/ 36/ 18	46	Vaginal (3.2 kg)/Walking at 14 m/o	n/a	n/a
ASD027/M	2009	22	92	3	22/12	47	18/ 21/ 39/ 17/ 12	23	C-section (2.4 kg)/Monozygotic twins, the sibling with ASD, walking at 15 m/o and babbling at 16 m/o, language and social regression, ADHD	n/a	n/a
ASD028/M	2007	38	113	3	20/12	42	16/ 24/ 36/ 22/ 6	18	Vaginal (3.3 kg), mother was 37 y/o/Language regression after 14 m/o	n/a	NAD
ASD029/M	2012	48	57	3	23/12	49	17/ 24/ 39/ 24/ 13	41	C-section (3.2 kg)/Walking at 13-14 m/o, ID	n/a	ABN

Proband/ Gender	DOB	Diagnosis							Clinical features		
		Age when diagnosed (month)		DSM-V level	ADOS score	CARS score	Denver II (month)		Delivery (weight at birth)/ Milestones & other presentations	Brain imaging	
		First time	Enrolling				PS/FM/GM/U/LA	DQ (%)		FDG-PET/CT (FDG hypometabolism)	MRI
ASD030/F	2010	22	79	2	22/12	37	18/ 48/ 54/ 48/ 42	53	C-section (3.8 kg)/Walking and babbling at 12 m/o; Language regression after 18 m/o	n/a	NAD
ASD031/M	2011	20	75	3	22/12	55	18/ 23/ 30/ 20/ 11	27	Vaginal (3.8 kg)/Walking at 12 m/o	Parietal lobe, central sulcus, temporal lobe, hippocampus, anterior cingulate gyrus	n/a
ASD032/M	2010	14	85	2	17/10	42	42/ 60/ 84/ 54/ 39	66	Vaginal (3.8 kg)/Walking at 13 m/o, seizure at 4 y/o, constipation	n/a	n/a
ASD033/M	2010	24	76	2	23/13	45	42/ 42/ 48/ 20/ 24	46	C-section (3.5 kg)/Babbling at 10 m/o, walking at 12 m/o, language regression after 12 m/o	n/a	n/a
ASD034/M	2011	21	77	3	18/12	50	41/ 24/ 63/ 20/ 24	45	Vaginal (3.3 kg)/Babbling at 9 m/o, walking at 14 m/o, language regression after 17 m/o	Hippocampus, anterior cingulate gyrus	NAD
ASD035/M	2009	36	99	3	20/12	45	27/ 17/ 39/ 19/ 15	24	Forceps delivery/Walking at 17 m/o, babbling at 24 m/o	n/a	ABN
ASD036/F	2009	18	57	3	20/12	49	42/ 42/ 72/ 18/ 15	66	Vaginal (3.5 kg)/DD, ADHD	n/a	NAD
ASD037/M	2012	24	52	3	52	51	11/ 15/ 36/ 12/ 8	32	Vaginal (3.2 kg), preterm birth at 32 weeks of gestation/Babbling at 10 m/o and walking at 16 m/o	n/a	n/a
ASD038/M	2010	24	48	n/a	22/12	48	6.5/ 9/ 15/ 10/ 8	20	C-section (2.6 kg)/Missed rolling and clawing, walking at 2 y/o; EP, ID	n/a	NAD
ASD039/M	2010	18	81	3	22/12	53	12/ 15/ 24/ 11/ 7	17	Vaginal (3.5 kg)/Babbling at 12 m/o, walking at 15 m/o	n/a	NAD
ASD040/M	2011	20	70	3	22/12	52	23/ 36/ 70/ 25/ 18	49	Vaginal (3.2 kg)/Walking and babbling at 12 m/o, language regression after 18 m/o	Parietal lobe, anterior cingulate gyrus, hippocampus	n/a
ASD041/M	2003	36	169	3	21/12	49	39/ 42/ 66/ 16/ 15	21	C-section (3.6kg)/Walking at 17-18 m/o, speech delay	n/a	n/a
ASD042/M	2011	36	71	3	22/12	48	15/ 17/ 36/ 11/ 12	26	Vaginal (3 kg)/Walking at 18 m/o, speech delay	n/a	n/a
ASD043/M	2003	36	166	2	22/12	44	16/ 18/ 24/ 18/ 12	11	Vaginal (3.3 kg)/Walking at 15 m/o, sensation disorders	n/a	n/a

Proband/ Gender	DOB	Diagnosis							Clinical features		
		Age when diagnosed (month)		DSM-V level	ADOS score	CARS score	Denver II (month)		Delivery (weight at birth)/ Milestones & other presentations	Brain imaging	
		First time	Enrolling				PS/FM/GM/U/LA	DQ (%)		FDG-PET/CT (FDG hypometabolism)	MRI
ASD044/M	2012	24	60	3	20/12	53	8/ 11/ 14/ 5/ 5	14	C-section (3.2 kg)/Missed crawling, walking at 21 m/o, language regression after 7-8 m/o, seizure at 12 m/o	Parietal lobe, central sulcus, temporal lobe, hippocampus, anterior cingulate gyrus	NAD
ASD045/M	2011	27	64	3	24/12	50	17/ 24/ 24/ 20/ 13	30	Vaginal (3.4 kg)/Walking at 14 m/o, speech delay	Temporal lobe, hippocampus, anterior cingulate gyrus	ABN
ASD046/M	2006	24	152	3	21/12	46	66/ 66/ 72/ 48/ 30	37	Vaginal (3.2 kg)/Babbling at 18-20 m/o, walking at 3 y/o, language regression after 2 y/o, motor delay, ID	n/a	NAD
ASD047/M	2009	18	90	3	22/12	50	17/ 17/ 23/ 14/ 10	18	C-section (2.9 kg)/Babbling at 7-8 m/o, walking at 28 m/o	n/a	NAD
ASD048/M	2007	18	119	2	20/12	40	33/ 36/ 48/ 22/ 13	26	Vaginal (3.7 kg)/Walking at 13 m/o, sensation disorders	n/a	n/a
ASD049/M	2007	24	115	3	21/12	46	66/ 36/ 108/ 36/ 20	46	C-section (3.7 kg)/Walking at 12-13 m/o, language regression after 18 m/o	n/a	n/a
ASD050/M	2011	36	66	3	22/12	52	11/ 15/ 42/ 15/ 11	28	C-section (3 kg), mother was 40 y/o when delivered/Babbling at 11 m/o, walking at 17 m/o	n/a	n/a
ASD051/F	2007	24	112	1-2	n/a	35	72/ 54/ 72/ 42/ 36	49	Vaginal (2.6 kg)/Walking at 12 m/o, babbling at 24 m/o	n/a	ABN
ASD052/M	2014	18	30	2	20/12	n/a	20/ 22/ 24/ 12/ 15	62	Vaginal (3.7 kg)/Walking at 13 m/o, talking at 30 m/o, DD	n/a	n/a
ASD053/M	2010	30	86	3	21/12	46	60/ 45/ 60/ 33/ 25	52	Vaginal (3.5 kg)/Walking and babbling at 11 m/o	n/a	n/a
ASD054/M	2011	24	76	3	16/12	50	15/ 15/ 24/ 15/ 10	21	C-section (2.7 kg), mother was infected with rubella at 4th months of gestation/Walking at 18 m/o, babbling at 36 m/o, sleep deprivation, ID	n/a	ABN
ASD056/M	2007	36	119	2	17/12	40	60/ 72/ 72/ 57/ 42	51	Vaginal (3.4 kg)/Walking at 17-18 m/o and babbling at 24 m/o, language regression after 24 m/o	n/a	NAD
ASD057/M	2011	36	70	3	20/12	48	24/ 30/ 48/ 33/ 18	44	C-section (2.5 kg)/Walking at12 m/o, speech delay	n/a	n/a

Proband/ Gender	DOB	Diagnosis							Clinical features		
		Age when diagnosed (month)		DSM-V level	ADOS score	CARS score	Denver II (month)		Delivery (weight at birth)/ Milestones & other presentations	Brain imaging	
		First time	Enrolling				PS/FM/GM/U/LA	DQ (%)		FDG-PET/CT (FDG hypometabolism)	MRI
ASD058/M	2012	24	59	3	20/12	50	24/ 30/ 48/ 33/ 18	52	Vaginal (4.2 kg)	n/a	n/a
ASD059/M	2010	22	80	2-3	21/12	46	36/ 51/ 81/ 48/ 30	62	C-section (3 kg)/Sensation disorders, ID	n/a	NAD
ASD060/M	2012	16	54	2	15/12	38	36/ 42/ 54/ 36/ 42	78	Vaginal (3.4 kg)/Walking at 12 m/o, talking at 24 m/o	n/a	n/a
ASD061/M	2013	18	49	3	21/12	45	24/ 30/ 36/ 20/ 10	49	C-section (3.4 kg)/Walking at 14 m/o, babbling at 9 m/o, language regression after 12 m/o	n/a	n/a
ASD062/M	2011	28	74	3	18/12	46	14/ 24/ 36/ 12/ 6.5	24	Vaginal (3.3 kg)/2nd child, older brother presented speech delay, walking at 12 m/o, sleep disorder	Parietal lobe, central sulcus, hippocampus, anterior cingulate gyrus	n/a
ASD063/M	2012	36	56	3	20/12	45	21/ 27/ 36/ 23/ 17	44	C-section (4.1 kg)/Walking at 15 m/o	Hippocampus, frontal lobe	NAD
ASD064/M	2007	24	117	2	15/12	40	54/ 51/ 51/ 26/ 13	33	C-section (3.4 kg)/Walking at 14-15 m/o, speech delay	n/a	n/a
ASD065/F	2010	n/a	82	2-3	22/12	43	18/ 24/ 30/ 24/ 16	27	C-section (3.35 kg)/Older brother with CP, walking and babbling at 12 m/o, language regression after 12 m/o, EP at 8 m/o, sensation disorders, DD	n/a	n/a
ASD066/M	2012	24	52	3	21/12	50	18/ 20/ 48/ 16/ 18	46	C-section (2.9 kg), mother was 43 y/o when delivered/Walking at 16 m/o	n/a	n/a
ASD067/M	2009	18	98	3	22/12	46	30/ 36/ 60/ 30/ 24	37	Vaginal (3.2 kg)/Walking at 14 m/o, babbling at 20 m/o, sleep disorder	n/a	n/a
ASD068/F	2013	18	44	3	20/12	54	4.5/ 9/ 16/ 6/ 9	20	Vaginal (4.2 kg)/Walking at 12 m/o, seizure	n/a	NAD
ASD069/M	2011	24	73	3	22/12	49	18/ 29/ 31/ 17/ 14	29	Vaginal (3.1 kg), mother was 39 y/o when delivered/Walking at 15 m/o, talking at 48 m/o	Hippocampus, anterior cingulate gyrus	NAD
ASD070/M	2013	18	42	3	20/12	49	11/ 24/ 24/ 15/ 15	41	Vaginal (2.9 kg)/Walking and babbling at 12 m/o, language regression after 16 m/o	Central sulcus, hippocampus	n/a
ASD071/M	2012	36	59	3	20/12	53	9.5/ 19/ 29/ 11/ 5.5	25	C-section (3 kg), mother was under depression treatment during pregnancy/Walking at 22 m/o, babbling at 12 m/o, language regression after 18 m/o	Central sulcus, hippocampus, anterior cingulate gyrus	NAD

Proband/ Gender	DOB	Diagnosis							Clinical features		
		Age when diagnosed (month)		DSM-V level	ADOS score	CARS score	Denver II (month)		Delivery (weight at birth)/ Milestones & other presentations	Brain imaging	
		First time	Enrolling				PS/FM/GM/U/LA	DQ (%)		FDG-PET/CT (FDG hypometabolism)	MRI
ASD072/M	2012	24	59	3	20/12	56	13/ 21/ 36/ 8.5/ 8.5	29	Vaginal (1.3 kg), preterm birth at 7th month of gestation/Older brother with speech delay; Walking at 15 m/o, babbling at 18 m/o, asthma	Parietal lobe, hippocampus, anterior cingulate gyrus	NAD
ASD073/F	2012	24	55	3	20/12	50	23/ 24/ 36/ 15/ 7.5	38	C-section (3.9 kg)/Walking at 9 m/o	Temporal lobe, hippocampus, anterior cingulate gyrus	n/a
ASD074/M	2009	21	95	2-3	21/12	39	30/ 54/ 48/ 36/ 24	40	Vaginal (3.4 kg) at 37 weeks of gestation/Visual defect, walking at 18 m/o, talking at 12 m/o, sleep deprivation	n/a	ABN
ASD075/M	2012	24	59	3	19/12	51	16/ 25/ 36/ 12/ 10	33	C-section (3.1 kg), birth asphyxia/Older brother with speech delay and ID	Hippocampus, parietal lobe, anterior cingulate gyrus	n/a
ASD076/M	2011	24	70	3	20/12	54	26/ 25/ 39/ 15/ 8.5	32	C-section (3.7 kg)/Walking at 14 m/o, babbling at 12 m/o, language regression	Hippocampus, frontal lobe	n/a
ASD077/M	2011	30	71	2-3	18/12	42	24/ 33/ 30/ 20/ 13	34	C-section at 41 weeks of gestation (3.6 kg)/Walking at 13 m/o, babbling at 24 m/o	Hippocampus, anterior cingulate gyrus	n/a
ASD078/M	2014	18	36	3	16/12	45	15/ 24/ 24/ 11/ 12	48	Emergency C-section at 38 weeks of gestation (3.5 kg) due to intrauterine growth restriction	n/a	n/a
ASD079/M	2011	20	70	3	24/12	51	24/ 31/ 39/ 22/ 14	37	C-section (3.6 kg)/Walking at 12 m/o	Parietal lobe, hippocampus, anterior cingulate gyrus	n/a
ASD080/F	2012	48	66	3	21/12	50	18/ 31/ 36/ 15/ 14	34	Vaginal/Walking at 2 y/o	Frontal lobe, anterior cingulate gyrus, hippocampus	n/a
ASD081/M	2013	19	54	3	20/12	49	25/ 31/ 39/ 22/ 15	49	C-section at 37 weeks of gestation (3.7 kg)/Walking at 13 m/o, talking at 13 m/o, language regression after 17-18 m/o	Hippocampus, anterior cingulate gyrus	n/a
ASD082/F	2012	24	62	3	n/a	45	16/ 24/ 24/ 16/ 15	30	C-section (2.9 kg)/Congenital foot deformity, walking at 19-20 m/o	n/a	n/a

Proband/ Gender	DOB	Diagnosis							Clinical features		
		Age when diagnosed (month)		DSM-V level	ADOS score	CARS score	Denver II (month)		Delivery (weight at birth)/ Milestones & other presentations	Brain imaging	
		First time	Enrolling				PS/FM/GM/U/LA	DQ (%)		FDG-PET/CT (FDG hypometabolism)	MRI
ASD083/M	2011	26	50	2-3	20/12	46	19/ 23/ 36/ 16/ 12	42	C-section (3.2 kg)/Walking and babbling at 9 m/o	n/a	n/a
ASD084/M	2013	24	53	3	22/12	46	22/ 42/ 48/ 28/ 18	60	Vaginal (3.3 kg)/Walking at 13 m/o, babbling at 18 m/o, language delay after 18 m/o	n/a	n/a
ASD085/M	2013	18	42	3	20/12	44	17/ 19/ 18/ 18/ 10	39	Vaginal at 35 weeks of gestation (2.4 kg)/Walking at 12 m/o, ID	n/a	NAD
ASD086M	2012	17	57	2-3	21/12	45	13/ 24/ 30/ 10/ 9	30	C-section (4 kg)/Walking at 12 m/o, speech delay	n/a	n/a
ASD087/M	2010	36	88	3	23/12	44	24/ 48/ 54/ 36/ 16	40	Vaginal (3.3 kg)	n/a	ABN
ASD088/M	2014	39	41. 5	2-3	20/12	39	18/ 19/ 36/ 18/ 22	54	C-section at 36 weeks of gestation (3.6 kg)/Walking at 13 m/o, babbling at 9 m/o, often constipation	n/a	n/a
ASD089/M	2012	36	62	3	20/12	54	6.5/ 19/ 16/ 5.5/ 5.5	17	Vaginal (3.3 kg)/Walking at 24 m/o, seizure at 3 y/o, ID	n/a	ABN
ASD090/M	2010	24	80	3	18/12	49	4.5/ 3/ 72/ 30/ 30	35	Vaginal (3.5 kg)/Walking at 10 m/o	Anterior cingulate gyrus, hippocampus	n/a
ASD091/F	2009	21	99	3	22/12	51	9/ 15/ 24/ 11/ 6	13	Vaginal preterm birth at 36 weeks of gestation (3.8 kg)	n/a	NAD
ASD092/M	2012	24	63	3	19/12	53	25/ 25/ 36/ 11/ 5.5	32	C-section (3.9 kg)/Walking at 14 m/o, babbling at 10 m/o, language regression	n/a	ABN
ASD093/M	2008	22	109	3	19/12	45	21/ 25/ 42/ 18/ 10	21	C-section (3 kg)/Walking at 17 m/o, babbling at 18-19 m/o	n/a	NAD
ASD094/M	2009	30	95	1-2	15/12	41	60/ 42/ 30/ 39/ 27	42	Vaginal, no concern at birth	n/a	n/a
ASD095/M	2012	18	62	3	22/12	53	12/ 17/ 21/ 12/ 12	24	Vaginal (3 kg)/Chronic constipation, malnutrition	Central sulcus, hippocampus, anterior cingulate gyrus	n/a
ASD096/M	2009	26	87	3	22/12	48	24/ 39/ 87/ 24/ 12	43	C-section (3.6 kg)/Normal motor milestones (crawling, walking), high fever and seizure at 3 m/o	n/a	NAD
ASD097/M	2004	24	154	3	22/12	50	10/ 12/ 20/ 9/ 6.5	8	Vaginal (3.2 kg), mother was 35 y/o/Walking at 24 m/o, speech delay, sleep deprivation, ID	n/a	NAD

Proband/ Gender	DOB	Diagnosis							Clinical features		
		Age when diagnosed (month)		DSM-V level	ADOS score	CARS score	Denver II (month)		Delivery (weight at birth)/ Milestones & other presentations	Brain imaging	
		First time	Enrolling				PS/FM/GM/U/LA	DQ (%)		FDG-PET/CT (FDG hypometabolism)	MRI
ASD098/F	2009	18	88	n/a	n/a	41	27/ 36/ 88/ 36/ 30	49	Vaginal (3.2 kg), full term/Rolling at 5 m/o, walking at 15 m/o, babbling at 14 m/o, language and social interaction regressed after 16 m/o	n/a	ABN
ASD099/M	2008	24	66	3	21/12	48	30/ 33/ 36/ 30/ 15	44	Vaginal at 38 weeks of gestation (3.7 kg)/Walking at 14 m/o, babbling at 12 m/o, sleep disorder, Chiari I	n/a	ABN
ASD100/F	2008	36	95	n/a	n/a	48	18/ 42/ 72/ 21/ 14	35	C-section (3.4 kg), mother was 36 y/o/Walking at 12 m/o	n/a	NAD
ASD101/M	2011	24	68	3	n/a	48	13/ 18/ 17/ 9/ 6	19	Vaginal, birth asphyxia/Walking at 30 m/o, ID	Hippocampus, anterior cingulate gyrus	n/a
ASD102/M	2014	19	35	2-3	22/12	44	17/ 24/ 21/ 21/ 21	59	C-section (2.6 kg) at 37 weeks of gestation/Walking at 19 m/o, speech delay	Hippocampus, anterior cingulate gyrus	n/a
ASD103/M	2009	36	96	2-3	22/12	43	42/ 48/ 48/ 24/ 30	40	C-section (3.1 kg)/Walking at 12 m/o, speech delay	n/a	NAD

ABN (Abnormality); DQ (Developmental Quotient); M (Male); F (Female); m/o (months old); FM (Fine motor); GI (Gastrointestinal); GM (Gross motor); LA (Language); n/a (Not available); NAD (no abnormality detected); y/o (years old); PS (Personal-social); U (Understanding).

Supplementary Table S2. Whole exome sequencing quality

Proband (Gender)	Reads		Coverage on targeted region					
	Total Reads	Q30	Average depth (X)	1X	10X	20X	30X	50X
ASD002 (M)	128,399,220	95.4	101.4	99.4	95.2	89.2	82.2	66.7
UM	132,400,540	95.2	95.9	98.7	94.6	89.3	82.9	67.1
UF	108,918,172	93.8	68.1	98.9	94.4	85.9	74.2	47.8
ASD003 (M)	100,999,290	95.4	80.1	99.4	93.7	85.4	75.9	55.3
UM	104,394,399	93.9	69.5	98.9	93.5	84.6	73.1	48.1
UF	108,388,269	94.3	67.1	98.9	93.4	83.9	71.8	45.9
ASD004 (M)	103,025,414	95.4	86.6	99.4	94.0	86.3	77.6	58.9
UM	126,735,093	95.4	80.0	98.9	94.4	87.3	77.9	55.7
UF	127,696,515	93.6	79.7	99.0	94.7	87.7	78.2	55.8
AS (M)	116,366,212	94.9	82.4	98.9	94.5	87.7	79.2	59.4
ASD005 (M)	119,444,512	95.5	91.1	99.5	94.7	87.5	79.3	61.2
UM	76,597,832	95.9	63.4	98.4	88.9	77.3	65.2	42.3
UF	102,959,584	94.5	63.8	98.9	93.2	83.0	70.0	43.2
ASD006 (F)	121,989,124	95.4	94.8	99.3	94.6	87.8	80.1	62.7
UM	120,062,010	94.2	78.3	98.8	94.2	86.9	77.2	54.9
UF	136,859,866	94.6	81.8	99.0	94.6	87.4	78.1	56.6
ASD008 (F)	122,445,986	95.2	95.6	99.3	94.6	87.9	80.4	63.4
UM	115,418,119	94.7	73.7	98.9	93.8	85.5	75.0	51.1
UF	108,438,536	95.2	74.7	99.2	94.8	87.5	78.2	56.9
ASD009 (M)	135,468,066	96.1	100.5	99.5	95.1	88.3	80.4	62.6
UM	115,084,391	96.1	79.2	99.0	94.5	87.4	78.0	55.5
UF	112,128,646	95.6	70.5	98.9	93.5	84.2	72.5	48.1
ASD010 (M)	106,430,834	96.1	80.6	99.4	93.6	84.7	74.6	53.0
UM	130,331,711	95.6	88.0	99.0	94.6	87.9	79.4	59.8
UF	149,041,316	95.7	90.7	99.1	95.0	88.4	80.2	61.0
ASD011 (M)	130,710,926	95.1	88.6	98.9	94.7	88.6	80.8	62.3
UM	130,210,366	94.9	89.8	98.8	94.7	88.9	81.5	63.5
UF	138,028,264	94.8	96.8	99.0	95.5	90.4	83.7	67.2
ASD012 (M)	117,956,496	96.1	93.6	99.5	94.9	87.5	79.0	59.7
UM	114,699,354	95.8	74.7	98.9	93.3	84.6	74.0	51.0
UF	125,150,039	95.2	79.8	99.0	94.2	86.3	76.4	54.5
ASD013 (M)	123,202,964	96.3	91.3	99.5	94.5	86.7	77.6	57.4
UM	136,529,470	95.4	87.5	98.9	94.9	88.7	80.6	60.9
UF	108,064,608	95.5	69.5	98.9	93.4	84.0	72.4	47.9
ASD014 (M)	137,071,550	96.1	101.1	99.5	95.0	88.1	80.2	62.2
UM	116,956,184	95.4	78.6	98.8	93.7	85.8	76.0	54.1
UF	137,352,697	95.7	86.5	99.0	95.1	88.7	80.1	59.2
ASD015 (M)	125,956,416	96.0	95.7	99.5	94.8	87.6	79.1	59.9
UM	112,099,703	95.6	72.9	98.8	93.1	84.3	73.5	49.9
UF	118,464,529	95.1	76.8	99.0	94.2	86.4	76.3	53.4
ASD016 (M)	138,751,130	96.3	103.6	99.6	95.6	89.3	81.8	64.3
UM	132,844,912	94.5	84.5	98.8	95.0	89.2	81.0	59.9
UF	102,390,727	94.6	64.5	98.8	93.1	83.2	70.6	44.4
ASD017 (F)	110,682,760	96.4	85.5	99.3	93.9	85.8	76.2	54.8
UM	87,464,969	94.7	59.3	98.5	91.1	79.4	65.7	39.7
UF	158,586,861	94.0	92.6	98.9	95.6	90.4	82.9	63.1
ASD018 (M)	119,252,526	96.3	94.7	99.6	94.9	87.6	78.9	58.9
UM	92,273,246	95.1	61.9	98.6	91.9	81.5	68.7	42.3
UF	122,749,011	94.0	76.9	98.9	94.4	87.0	77.0	53.9
ASD019 (M)	127,457,474	96.5	94.2	99.6	95.0	87.7	79.1	59.4

Proband (Gender)	Reads		Coverage on targeted region					
	Total Reads	Q30	Average depth (X)	1X	10X	20X	30X	50X
UM	109,038,466	94.5	66.6	98.7	93.6	85.2	74.0	48.5
UF	87,493,910	94.4	52.1	98.6	91.2	78.2	62.5	33.9
ASD020 (M)	132,643,782	96.4	99.0	99.5	95.0	87.9	79.6	60.7
UM	96,824,056	94.0	60.6	98.6	92.6	82.5	69.4	42.2
UF	117,677,904	94.5	67.1	98.8	93.3	84.2	72.5	47.2
ASD021 (M)	124,490,314	96.3	95.6	99.5	94.4	86.8	78.0	58.2
UM	128,900,810	94.0	75.9	98.7	94.7	88.1	78.3	54.3
UF	132,250,637	93.2	78.4	98.8	95.2	88.6	78.3	54.3
ASD022 (M)	135,055,116	96.2	105.4	99.6	95.6	89.3	81.9	64.8
UM	100,069,598	95.4	68.0	99.0	93.7	84.9	73.8	48.7
UF	121,500,560	94.4	75.5	99.0	95.2	88.3	78.0	53.6
ASD023 (M)	101,123,124	96.1	79.3	99.4	93.5	84.5	74.1	51.8
UM	142,534,329	94.4	93.1	99.0	95.7	90.7	83.5	64.7
UF	124,230,647	95.1	76.3	99.1	94.6	87.0	77.0	54.1
ASD024 (F)	117,359,200	96.1	96.4	99.4	95.0	88.3	80.3	61.7
UM	110,944,491	95.3	73.7	99.0	94.2	86.4	76.1	52.4
UF	104,344,657	94.8	67.4	98.9	93.1	83.4	71.5	46.8
ASD025 (F)	122,453,792	94.9	90.0	98.8	94.4	88.1	80.5	62.5
UM	111,340,908	94.7	79.6	98.7	93.8	86.6	77.4	56.6
UF	126,853,428	94.4	94.1	98.9	95.2	89.9	83.0	66.1
ASD026 (M)	130,369,326	96.1	95.4	99.5	95.1	88.1	79.8	60.9
UM	110,509,359	95.3	72.1	98.7	93.3	84.8	74.2	50.7
UF	95,461,230	95.2	60.7	98.7	91.7	80.5	67.1	40.9
ASD027 (M)	125,090,600	96.2	96.8	99.6	94.8	87.5	78.9	59.7
UM	116,439,825	94.9	77.4	98.8	93.6	85.5	75.6	53.6
UF	130,858,728	95.0	79.0	99.0	94.4	86.8	77.1	54.9
AS (M)	128,240,938	95.9	98.4	99.5	95.1	88.1	80.1	61.8
ASD028 (M)	97,814,540	95.5	72.9	98.8	91.9	82.5	71.9	50.1
UM	142,853,236	94.6	100.2	98.9	95.4	90.7	84.8	69.7
UF	102,235,796	95.2	77.6	98.7	92.3	83.5	73.5	52.4
ASD029 (M)	137,571,233	94.8	86.1	99.0	94.9	88.4	79.8	58.9
UM	116,025,408.0	95.2	77.9	99.0	94.9	88.9	81.3	62.7
UF	96,265,434.0	95.1	62.6	99.0	93.8	85.4	74.8	50.1
ASD030 (F)	124,937,741	94.4	77.8	98.8	93.9	86.3	76.4	54.0
UM	113,646,538.0	94.8	80.2	99.1	95.4	89.9	82.8	65.0
UF	108,910,158	94.5	73.2	98.7	94.6	88.5	80.4	59.4
ASD031 (M)	102,934,718	94.4	67.3	99.0	93.3	84.0	72.4	47.0
UM	95,808,994	94.1	61.9	98.4	93.7	86.5	76.3	49.3
UF	118,491,230.0	95.2	78.3	99.2	95.3	89.2	81.4	62.6
ASD032 (M)	103,199,708	95.1	69.4	99.0	93.9	85.3	73.9	48.4
UM	140,822,280	94.6	88.2	99.1	96.4	92.0	85.7	68.1
UF	83,775,912	94.9	54.0	99.2	92.9	81.6	67.1	37.5
ASD033 (F)	114,232,777	94.2	71.7	98.8	93.6	85.0	74.0	49.4
UM	110,656,828	94.8	77.9	99.2	95.7	89.9	82.2	62.2
UF	107,966,430.0	96.7	73.8	98.9	94.0	86.2	76.6	54.4
ASD034 (M)	229,085,476	95.0	146.7	99.4	97.7	95.5	92.5	84.5
UM	128,400,158	94.0	79.9	99.2	96.4	91.4	84.1	63.8
UF	142,480,708	94.4	87.0	99.2	96.5	92.1	85.7	68.3
ASD035 (M)	88,937,746	94.4	53.6	99.1	93.7	83.3	69.3	38.7
UM	102,756,664	94.7	69.8	99.1	94.5	87.4	78.2	55.6
UF	113,288,024	94.7	68.5	99.2	95.1	87.8	78.1	54.3

Proband (Gender)	Reads		Coverage on targeted region					
	Total Reads	Q30	Average depth (X)	1X	10X	20X	30X	50X
ASD036 (F)	108,405,480	94.8	68.9	99.1	94.2	86.5	76.7	53.5
UM	123,441,310	94.2	76.2	99.2	95.9	90.5	82.9	62.1
UF	119,782,514	94.5	74.5	99.1	95.7	90.0	81.5	58.9
ASD037 (M)	92,262,204	94.5	56.3	99.0	93.8	84.0	70.5	40.6
UM	111,584,012	94.5	74.7	99.0	95.5	89.7	81.6	59.9
UF	85,460,986	95.1	54.3	99.0	91.8	80.1	65.8	37.1
ASD038 (M)	152,245,734	94.6	93.1	99.3	96.4	91.9	85.6	69.0
UM	116,416,804	93.9	69.5	99.0	95.3	88.9	79.4	54.4
UF	132,775,568	94.5	81.4	99.3	96.1	90.7	83.2	63.7
ASD039 (M)	93,125,344	94.7	58.7	99.2	93.4	83.4	70.7	43.5
UM	104,242,998	94.4	69.6	99.1	94.9	87.9	78.6	55.3
UF	110,955,214	94.7	66.7	99.3	94.6	86.3	75.6	50.6
ASD040 (M)	122,858,032	94.9	79.0	99.0	95.1	89.0	80.7	60.0
UM	98,697,012	95.1	64.4	98.8	93.4	84.8	73.6	48.3
UF	114,816,802	94.8	77.7	99.0	95.0	88.7	80.2	59.2
ASD041 (M)	117,653,916	93.8	69.3	99.2	96.0	89.7	80.2	54.9
UM	116,585,812	94.0	70.0	99.1	95.1	88.3	78.9	54.9
UF	104,755,632	93.9	71.7	98.6	94.6	88.5	80	57.9
ASD042 (M)	121,492,782	94.3	75.5	98.7	94.7	89.0	81.1	60.4
UM	116,971,856.0	96.6	86.2	99.5	96.7	91.9	85.3	68.4
UF	124,969,312.0	95.3	78.6	99.1	95.1	89.0	81.1	61.8
ASD043 (M)	138,757,064.0	94.5	86.9	99.0	96.4	92.6	86.6	69.2
UM	167,330,914.0	95.7	109.0	99.6	98.2	96.0	92.2	79.8
UF	128,496,732	95.2	85.0	99.1	94.7	88.2	80.4	61.9
ASD044 (M)	87,759,260	94.8	56.5	98.9	92.8	82.4	69.2	40.9
UM	107,773,612	94.8	74.6	98.9	94.3	87.5	78.8	57.7
UF	107,389,294	95.0	67.6	99.0	93.9	85.5	75.0	50.8
ASD045 (M)	115,783,268	94.7	73.6	99.0	94.6	87.6	78.3	56.0
UM	136,100,388	93.9	83.3	99.0	95.9	91.4	84.7	66.0
UF	116,166,472	94.9	75.8	99.2	95.0	88.2	79.4	58.1
ASD046 (M)	128,575,410	94.9	77.8	99.1	95.5	89.5	81.0	59.5
UM	119,721,904	94.9	75.1	99.1	95.0	88.2	79.0	56.6
UF	117,432,872	94.9	75.7	99.1	94.9	87.8	78.5	56.6
ASD047 (M)	92,377,186	95.0	60.0	99.0	92.6	82.1	69.6	43.4
UM	111,601,324	94.6	75.9	99.1	94.5	87.5	78.6	57.3
UF	131,286,196	94.3	82.8	98.7	95.1	90	83	64.4
ASD048 (M)	123,385,548	94.4	76.5	99.2	95.4	88.9	80.0	57.9
UM	132,212,132	94.3	82.8	99.2	95.6	90.0	82.6	63.5
UF	116,059,484	93.9	74.4	98.6	94.6	88.6	80.4	58.9
ASD049 (M)	115,181,068	92.8	70.8	98.8	94.7	88.5	80.2	58.9
UM	90,551,092	93	56.2	98.5	92.7	83.9	72.4	44.7
UF	116,982,228	92.7	76.5	98.8	95.1	89.7	82.5	63.6
ASD050 (M)	129,181,716	92.7	74.6	98.8	95.2	89.9	82.4	62.3
UM	120,355,850	92.9	73.2	98.6	94.5	88.9	81.4	61.4
UF	95,999,860	92.5	59.8	98.6	93.5	85.6	74.9	48.5
ASD051 (F)	111,901,734	95.6	75.8	99.0	93.5	85.4	75.8	54.9
UM	91,803,386	95.6	61.8	99.0	92.4	82.3	70.4	45.7
UF	123,268,970.0	95.1	80.9	99.1	95.6	90.1	82.4	62.8
ASD052 (M)	144,982,960	95.0	87.7	99.3	96.3	91.6	84.9	67.0
UM	128,750,308	95.1	81.7	99.0	95.4	89.8	82.3	63.2
UF	125,175,976	95.3	82.0	99.2	95.0	88.6	80.5	61.2

Proband (Gender)	Reads		Coverage on targeted region						
	Total Reads	Q30	Average depth (X)	1X	10X	20X	30X	50X
ASD053 (M)	108,012,998	94.4	71.1	98.8	94.8	88.3	79.3	56.1
UM	108,022,958	94.2	69.3	98.5	94.5	88.2	79.3	54.9
UF	114,977,162	93.8	76.8	98.8	95.4	90.2	82.4	60.8
ASD054 (M)	120,349,544	94.4	74.7	98.8	95.1	89.3	80.9	58.5
UM	122,649,626	94.2	77.3	98.7	95.1	89.8	82.1	60.3
UF	118,349,558	94	76.3	98.8	95	89.2	80.9	59.1
ASD056 (M)	140,290,284.0	96.9	116.6	99.2	94.7	89.1	83.1	70.6
UM	127,244,472.0	96.7	103.5	99.0	94.1	87.8	80.9	66.1
UF	123,931,280.0	96.7	105.5	99.1	94.2	87.7	80.7	65.8
ASD057 (M)	141,468,342.0	96.7	109.6	99.2	95.0	89.3	82.7	68.2
UM	123,830,642.0	96.4	97.6	99.3	95.4	88.7	80.9	63.7
UF	132,102,232.0	96.7	108.0	99.1	94.2	87.6	80.5	65.3
ASD058 (M)	120,613,040	94.7	77.6	99.1	95.4	89.7	82.1	62.3
UM	94,934,084	94.3	58.6	98.7	94.2	86.3	74.8	45.1
UF	104,363,528	94.3	68.9	98.9	94.9	88.2	78.9	55
ASD059 (M)	125,064,942	94.5	74.6	99.0	95.5	89.6	81.3	59.3
UM	126,886,006	94.2	74.9	98.9	95.4	89.9	81.8	59.1
UF	114,859,610	94	70.3	98.9	94.9	88.3	79.1	55.2
ASD060 (M)	142,646,278.0	96.7	104.7	99.5	97.1	93.1	87.9	74.6
UM	132,995,998.0	96.5	96.2	99.4	96.9	92.9	87.4	73.0
UF	104,311,976.0	96.4	80.6	99.4	96.5	91.1	83.7	64.9
ASD061 (M)	150,206,696.0	96.7	106.0	99.5	97.3	93.6	88.6	75.6
UM	120,054,694.0	96.3	78.8	99.0	95.5	90.0	82.2	62.0
UF	134,769,652.0	96.9	101.6	99.4	96.3	91.6	85.9	72.5
ASD062 (M)	110,712,548.0	95.6	79.6	99.5	96.4	90.8	83.3	64.3
UM	103,532,448.0	95.3	72.0	99.3	96.3	90.7	82.4	59.7
UF	119,135,514.0	94.8	85.6	99.5	97.3	93.3	86.9	68.8
ASD063 (M)	122,251,692.0	94.8	79.0	99.4	97.1	92.6	84.9	62.8
UM	144,208,308.0	96.1	88.4	99.1	96.4	92.2	85.8	68.3
UF	152,522,652.0	95.3	102.2	99.5	97.4	94.0	89.1	75.3
ASD064 (M)	113,914,448	94.9	81.1	99.1	95.2	89.3	81.4	61.8
UM	99,010,164	94.8	70	99	94.6	87.6	78.3	54.5
UF	130,448,588	94.4	95.1	99.1	96.4	92.4	86.8	71.3
ASD065 (F)	134,646,234.0	95.1	88.0	99.1	95.6	90.1	82.7	64.1
UM	124,198,212	94.9	85.1	99	95.6	90.6	83.8	65.8
UF	98,555,240	95	73.6	99.1	94.3	86.9	78	56.9
ASD066 (M)	102,060,050.0	95.5	69.0	99.2	93.5	83.7	72.0	47.8
UM	113,518,518.0	94.8	71.8	99.2	96.0	89.7	80.4	56.6
UF	108,402,284.0	94.5	72.5	99.3	96.3	89.9	80.6	56.9
ASD067 (M)	118,381,990	94.9	87.4	99.3	95.7	90.1	83.0	65.5
UM	95,390,476	94.8	69.5	99.1	94.4	86.9	77.3	53.5
UF	101,664,098	94.7	78.6	99.2	95	88.4	79.9	59.2
ASD068 (F)	121,461,306	95	85.9	99.2	95.2	89.4	82.1	63.8
UM	130,400,230	94.7	91.7	99.2	95.6	90.3	83.4	65.8
UF	127,035,564	94.3	89	99.3	95.8	90.1	82.5	63.6
ASD069 (M)	133,447,318	95	96.1	99.4	96.4	91.6	85.2	68.7
UM	129,921,862	95	93.4	99.3	96.3	91.9	85.8	69.4
UF	134,815,912	94.7	101.5	99.4	96.8	92.9	87.4	72.7
ASD070 (M)	155,866,618	94.9	105.3	99.4	97.0	93.3	88.1	74.0
UM	116,592,972.0	96.3	74.7	98.9	95.0	88.2	79.1	56.6
UF	130,560,378.0	94.8	77.8	99.4	96.8	91.3	82.9	60.8

Proband (Gender)	Reads		Coverage on targeted region						
	Total Reads	Q30	Average depth (X)	1X	10X	20X	30X	50X
ASD071 (M)	126,185,296.0	94.6	76.4	99.4	96.5	90.5	81.5	58.3
UM	139,647,024.0	94.6	86.2	99.3	96.9	92.5	85.6	66.2
UF	126,737,518.0	94.9	80.2	99.4	96.5	90.7	82.2	60.8
ASD072 (M)	97,175,008	94.1	66.8	99.3	95.8	88.9	79.0	53.3
UM	88,011,462	94	60.5	99	94.7	86.8	75.7	46.8
UF	79,389,712	94.1	58.5	99.2	93.6	84.1	72	44.1
ASD073 (F)	197,628,456	94.2	123.2	99.4	97.4	94.8	91.2	81.4
UM	102,084,794	94.3	71.2	98.9	94.8	88.4	79.6	55.7
UF	103,752,412.0	94.7	64.7	99.4	95.5	87.1	75.2	48.5
ASD074 (M)	123,998,808	94.3	83.2	99.4	96.8	92.4	85.8	67.4
UM	108,809,202	94.6	75.6	99.2	95.9	90.5	83.1	62.8
UF	99,689,478	94.3	71.1	99.4	96.0	90.0	81.4	58.7
ASD075 (M)	118,344,272.0	94.4	77.2	99.5	96.6	90.6	81.6	59.3
UM	134,124,374.0	95.0	81.1	99.4	96.8	91.5	83.6	62.9
UF	119,416,178	94.6	81.1	99.4	96.3	91.1	84.0	65.4
ASD076 (M)	116,373,858	94.4	80.0	99.4	96.1	90.7	83.5	65.0
UM	108,781,324	94.9	79.4	99.3	96.0	90.6	83.6	65.4
UF	102,612,692	94.8	74.0	99.4	95.5	89.1	81.0	60.5
ASD077 (M)	111,585,766	94.6	83.4	99.5	96.4	91.1	84.3	66.7
UM	128,039,400	94.8	86.3	99.4	96.5	91.8	85.6	69.0
UF	116,475,638	94.8	81.4	99.4	96.0	90.4	83.1	64.5
ASD078 (M)	113,327,118.0	95.1	71.7	99.4	95.5	88.0	77.7	53.9
UM	125,366,096.0	95.3	82.2	99.4	96.1	89.9	81.5	61.3
UF	170,310,164.0	95.2	119.5	99.2	95.0	89.1	82.1	67.0
ASD079 (M)	119,246,258	94.6	83.5	99.5	96.1	90.7	83.6	65.7
UM	107,800,426	95.1	70.7	99.1	94.5	87.4	78.4	56.2
UF	133,421,376	94.8	93.6	99.2	96	91.2	85	68.7
ASD080 (F)	143,834,870.0	95.3	102.0	99.0	93.8	86.4	77.9	60.2
UM	87,981,334.0	95.0	67.0	98.7	90.6	78.9	66.2	42.5
UF	138,748,812.0	95.4	95.4	99.0	93.5	85.3	76.2	57.3
ASD081 (M)	146,264,604	94.6	106.1	99.4	96.9	93.1	87.9	74.2
UM	104,296,468	93.7	68.8	99	95	88.2	78.7	53.7
UF	120,800,542	94.5	78.4	99.4	96.4	91.3	84.0	64.2
ASD082 (F)	128,733,768.0	96.7	104.7	99.2	94.6	88.2	81.1	66.0
UM	104,429,038.0	96.6	85.0	99.0	92.9	85.0	76.3	58.0
UF	151,362,186.0	96.5	124.8	99.3	95.6	90.4	84.7	72.1
ASD083 (M)	140,130,998.0	96.7	108.3	99.2	95.0	89.0	82.3	67.5
UM	110,332,122.0	96.9	89.5	99.0	92.0	83.2	74.1	55.6
UF	152,709,862.0	96.7	124.4	99.3	95.4	90.4	85.0	73.4
ASD084 (M)	129,279,458.0	96.9	106.7	99.3	94.1	87.3	80.1	65.0
UM	116,868,566.0	96.7	94.7	99.2	93.7	86.3	78.2	61.2
UF	132,256,906.0	96.4	109.0	99.4	95.3	89.4	82.7	67.9
ASD085 (M)	135,398,286.0	96.8	105.0	99.4	94.8	88.2	80.8	65.1
UM	126,433,720.0	96.6	99.6	99.3	94.7	87.9	80.2	63.6
UF	149,198,590.0	96.7	119.1	99.4	94.8	88.3	81.2	66.4
ASD086 (M)	106,746,606.0	96.8	89.3	99.2	93.2	85.0	76.1	57.6
UM	68,912,282.0	96.9	54.5	98.6	87.4	73.0	58.4	32.1
UF	101,115,156.0	96.8	88.5	99.2	92.9	84.6	75.7	57.4
ASD087 (M)	130,606,278.0	96.8	103.2	99.4	95.1	88.8	81.6	65.8
UM	104,995,788.0	96.8	85.8	99.0	92.2	83.3	73.9	54.2
UF	122,439,988.0	97.0	103.9	99.2	93.6	86.5	79.1	64.0

Proband (Gender)	Reads			Coverage on targeted region				
	Total Reads	Q30	Average depth (X)	1X	10X	20X	30X	50X
ASD088 (M)	104,047,350	96.5	75.8	99.2	93.7	85.5	75.9	54.6
UM	116,263,436.0	96.3	90.2	99.4	94.4	87.2	79.1	61.4
UF	85,975,658.0	95.8	66.1	99.3	92.3	81.9	70.3	46.8
ASD089 (M)	118,050,150.0	96.8	83.7	99.1	94.2	86.4	77.1	56.6
UM	113,631,238.0	95.1	73.0	99.0	95.2	88.7	79.5	56.2
UF	101,284,670	95.8	71.3	99.2	92.9	83.1	71.9	48.7
ASD090 (M)	124,672,174.0	97.0	109.0	99.3	92.3	82.9	73.3	54.2
UM	107,324,894.0	96.9	93.5	99.2	91.9	82.0	71.7	50.9
UF	141,317,122.0	96.8	124.7	99.5	95.1	88.8	81.9	67.2
ASD091 (F)	149,271,896.0	97.0	123.9	99.4	95.0	88.9	82.2	68.0
UM	125,535,086.0	97.1	107.6	99.2	92.7	84.2	75.6	58.5
UF	143,761,210.0	97.1	127.4	99.4	94.1	87.1	80.0	65.8
ASD092 (M)	142,001,080.0	96.8	121.6	99.6	95.0	88.7	81.9	67.8
UM	125,520,448.0	96.7	105.9	99.4	94.0	86.5	78.4	61.5
UF	129,046,526.0	96.6	113.0	99.5	94.6	87.6	79.9	63.9
ASD093 (M)	121,909,236.0	96.9	101.3	99.4	92.9	83.9	74.6	55.9
UM	107,423,912.0	96.9	91.0	99.2	91.5	81.6	71.4	51.1
UF	159,449,908.0	96.5	130.8	99.6	96.4	91.3	85.4	72.3
ASD094 (M)	117,267,964.0	96.4	91.6	99.6	94.4	86.5	77.6	58.9
UM	170,871,654.0	95.6	112.4	99.2	96.8	93.4	88.5	75.2
UF	74,666,352.0	96.4	63.0	99.3	89.9	77.3	64.4	39.8
ASD095 (M)	172,899,630.0	95.4	116.1	99.4	97.0	93.7	88.8	75.6
UM	138,617,798.0	94.9	89.9	99.1	96.3	91.9	85.5	68.1
UF	106,654,806.0	96.6	84.9	99.4	92.9	84.1	74.8	56.0
ASD096 (M)	126,295,222	95.6	82.6	99.3	95.8	89.9	81.8	61.6
UM	115,540,610	95.6	75.3	99.2	95.1	88.5	79.5	57.5
UF	115,836,232	95.5	79.3	99.3	95.7	89.3	80.7	59.7
ASD097 (M)	123,181,930	95.7	76.5	99.4	95.6	88.6	79.2	56.8
UM	112,663,448	95.7	72.8	99.2	94.5	86.6	76.5	53.2
UF	127,231,720	95.6	82.3	99.3	95.5	88.9	80.3	59.7
ASD098 (F)	112,452,698.0	95.1	74.8	99.0	95.3	88.9	80.1	58.0
UM	129,868,090	96.0	87.0	99.3	95.8	90.0	82.4	63.5
UF	129,207,094	95.9	90.5	99.4	95.9	89.9	82.2	63.4
ASD099 (M)	105,550,758	96.6	74.4	99.2	91.9	81.5	69.8	46.5
UM	137,829,810	96.0	91.1	99.2	95.6	89.9	82.5	64.1
UF	147,598,372	95.9	99.2	99.4	96.1	91.0	84.5	68.2
ASD100 (F)	129,479,222	96.3	87.9	99.2	95.6	89.6	81.9	63.1
UM	119,673,132	96.4	81.4	99.2	95.1	88.4	79.8	59.3
UF	126,226,812	96.2	88.8	99.4	95.9	89.8	82.1	63.3
ASD101 (M)	121,594,926	96.4	80.2	99.2	94.9	87.9	78.9	58.0
UM	133,609,320	96.2	87.1	99.2	95.7	89.7	81.7	62.3
UF	127,796,030	96.3	86.9	99.4	95.4	88.7	80.2	60.7
ASD102 (M)	138,253,426	95.8	92.6	99.4	95.7	89.8	82.3	64.4
UM	125,785,568	95.2	75.6	99.1	93.2	83.1	71.0	47.6
UF	145,972,688	95.5	100.1	99.4	96.6	92.1	85.9	70.1
ASD103 (M)	110,670,390	96.2	75.7	99.3	92.8	83.2	72.3	49.8
UM	138,728,820	95.7	90.1	99.3	96.1	90.8	83.9	66.1
UF	136,519,708.0	95.0	85.4	99.2	96.4	91.7	84.8	66.3

F (Female); M (Male); UF (Unaffected father); UM (Unaffected mother)

Supplementary Table S3. Relatedness analysis

sample_a	sample_b	shared_hets	hets_a	ibs2	rel	hets_b	ibs0	n	pedigree_parents	predicted_parents	parent_error	rel difference
ASD002_child	ASD002_mother	1079	2207	8254	0.481	2195	12	10509	TRUE	TRUE	FALSE	0.01936
ASD002_child	ASD002_father	1090	2207	8204	0.481	2275	14	10509	TRUE	TRUE	FALSE	0.0188
ASD003_child	ASD003_mother	1113	2238	8215	0.479	2273	21	10511	TRUE	TRUE	FALSE	0.02145
ASD003_child	ASD003_father	1081	2238	8197	0.466	2237	19	10511	TRUE	TRUE	FALSE	0.03375
ASD004_child	ASD004_mother	1196	2292	8281	0.51	2344	14	10528	TRUE	TRUE	FALSE	-0.009599
ASD004_child	ASD004_father	1091	2292	8152	0.469	2253	17	10528	TRUE	TRUE	FALSE	0.03085
ASD005_child	ASD005_mother	1089	2286	8107	0.473	2178	30	10416	TRUE	TRUE	FALSE	0.02755
ASD005_child	ASD005_father	1091	2286	8069	0.477	2234	13	10416	TRUE	TRUE	FALSE	0.02328
ASD006_child	ASD006_mother	1054	2198	8269	0.472	2176	14	10537	TRUE	TRUE	FALSE	0.02849
ASD006_child	ASD006_father	1093	2198	8194	0.479	2314	20	10537	TRUE	TRUE	FALSE	0.02093
ASD008_child	ASD008_mother	1105	2288	8213	0.474	2274	14	10571	TRUE	TRUE	FALSE	0.02639
ASD008_child	ASD008_father	1128	2288	8254	0.481	2267	19	10571	TRUE	TRUE	FALSE	0.01919
ASD009_child	ASD009_mother	1176	2350	8283	0.505	2245	21	10541	TRUE	TRUE	FALSE	-0.005122
ASD009_child	ASD009_father	1213	2350	8234	0.5	2374	19	10541	TRUE	TRUE	FALSE	0
ASD010_child	ASD010_mother	1115	2314	8289	0.498	2177	15	10559	TRUE	TRUE	FALSE	0.001608
ASD010_child	ASD010_father	1171	2314	8243	0.486	2337	23	10559	TRUE	TRUE	FALSE	0.01383
ASD011_child	ASD011_mother	1129	2239	8273	0.495	2281	10	10533	TRUE	TRUE	FALSE	0.00469
ASD011_child	ASD011_father	1097	2239	8291	0.483	2190	20	10533	TRUE	TRUE	FALSE	0.01735
ASD012_child	ASD012_mother	1106	2282	8262	0.486	2184	22	10541	TRUE	TRUE	FALSE	0.01374
ASD012_child	ASD012_father	1121	2282	8213	0.479	2285	14	10541	TRUE	TRUE	FALSE	0.02103
ASD013_child	ASD013_mother	1105	2282	8237	0.483	2221	16	10525	TRUE	TRUE	FALSE	0.01688
ASD013_child	ASD013_father	1136	2282	8209	0.482	2296	18	10525	TRUE	TRUE	FALSE	0.01797
ASD014_child	ASD014_mother	1080	2158	8334	0.482	2210	20	10554	TRUE	TRUE	FALSE	0.01807
ASD014_child	ASD014_father	1057	2158	8264	0.477	2234	14	10554	TRUE	TRUE	FALSE	0.02317
ASD015_child	ASD015_mother	1041	2209	8250	0.468	2136	21	10525	TRUE	TRUE	FALSE	0.0323
ASD015_child	ASD015_father	1035	2209	8177	0.454	2204	17	10525	TRUE	TRUE	FALSE	0.04583

sample_a	sample_b	shared_hets	hets_a	ibs2	rel	hets_b	ibs0	n	pedigree_parents	predicted_parents	parent_error	rel difference
ASD016_child	ASD016_mother	1202	2278	8240	0.519	2404	10	10530	TRUE	TRUE	FALSE	-0.01888
ASD016_child	ASD016_father	1099	2278	8224	0.48	2205	20	10530	TRUE	TRUE	FALSE	0.01973
ASD017_child	ASD017_mother	1066	2221	8177	0.465	2194	23	10470	TRUE	TRUE	FALSE	0.0351
ASD017_child	ASD017_father	1081	2221	8157	0.475	2258	13	10470	TRUE	TRUE	FALSE	0.02499
ASD018_child	ASD018_mother	1063	2281	8222	0.484	2125	17	10512	TRUE	TRUE	FALSE	0.01576
ASD018_child	ASD018_father	1139	2281	8158	0.484	2344	18	10512	TRUE	TRUE	FALSE	0.01644
ASD019_child	ASD019_mother	1094	2194	8264	0.485	2214	15	10494	TRUE	TRUE	FALSE	0.01504
ASD019_child	ASD019_father	1077	2194	8191	0.475	2239	17	10494	TRUE	TRUE	FALSE	0.02461
ASD020_child	ASD020_mother	1056	2293	8167	0.475	2154	17	10512	TRUE	TRUE	FALSE	0.02553
ASD020_child	ASD020_father	1159	2293	8209	0.478	2300	31	10512	TRUE	TRUE	FALSE	0.02159
ASD021_child	ASD021_mother	1111	2290	8254	0.485	2207	20	10533	TRUE	TRUE	FALSE	0.01473
ASD021_child	ASD021_father	1219	2290	8284	0.518	2390	17	10533	TRUE	TRUE	FALSE	-0.01747
ASD022_child	ASD022_mother	1109	2332	8182	0.475	2268	16	10566	TRUE	TRUE	FALSE	0.02513
ASD022_child	ASD022_father	1189	2332	8272	0.496	2337	16	10566	TRUE	TRUE	FALSE	0.003859
ASD023_child	ASD023_mother	1112	2234	8354	0.5	2174	13	10546	TRUE	TRUE	FALSE	0.00046
ASD023_child	ASD023_father	1129	2234	8291	0.496	2277	11	10546	TRUE	TRUE	FALSE	0.004476
ASD024_child	ASD024_mother	1106	2256	8274	0.488	2225	10	10544	TRUE	TRUE	FALSE	0.01191
ASD024_child	ASD024_father	1074	2256	8232	0.473	2193	18	10544	TRUE	TRUE	FALSE	0.02668
ASD025_child	ASD025_mother	1073	2186	8228	0.474	2230	19	10509	TRUE	TRUE	FALSE	0.02653
ASD025_child	ASD025_father	1102	2186	8271	0.486	2242	20	10509	TRUE	TRUE	FALSE	0.01418
ASD026_child	ASD026_mother	1088	2276	8155	0.474	2230	16	10505	TRUE	TRUE	FALSE	0.02646
ASD026_child	ASD026_father	1080	2276	8145	0.47	2224	17	10505	TRUE	TRUE	FALSE	0.02968
ASD027_child	ASD027_mother	1146	2315	8168	0.479	2331	19	10532	TRUE	TRUE	FALSE	0.02138
ASD027_child	ASD027_father	1109	2315	8178	0.48	2250	14	10532	TRUE	TRUE	FALSE	0.01956
ASD028_child	ASD028_mother	1123	2261	8106	0.476	2310	24	10446	TRUE	TRUE	FALSE	0.02455
ASD028_child	ASD028_father	1117	2261	8165	0.479	2241	22	10446	TRUE	TRUE	FALSE	0.0212
ASD029_child	ASD029_mother	1109	2251	8183	0.477	2282	18	10509	TRUE	TRUE	FALSE	0.02332

sample_a	sample_b	shared_hets	hets_a	ibs2	rel	hets_b	ibs0	n	pedigree_parents	predicted_parents	parent_error	rel difference
ASD029_child	ASD029_father	1085	2251	8182	0.469	2236	18	10509	TRUE	TRUE	FALSE	0.03086
ASD030_child	ASD030_mother	1122	2259	8204	0.478	2266	21	10498	TRUE	TRUE	FALSE	0.02191
ASD030_child	ASD030_father	1110	2259	8219	0.486	2230	13	10498	TRUE	TRUE	FALSE	0.0139
ASD031_child	ASD031_mother	1141	2288	8230	0.496	2247	13	10482	TRUE	TRUE	FALSE	0.003783
ASD031_child	ASD031_father	1120	2288	8135	0.469	2296	23	10482	TRUE	TRUE	FALSE	0.03059
ASD032_child	ASD032_mother	1073	2265	8175	0.466	2242	14	10543	TRUE	TRUE	FALSE	0.0339
ASD032_child	ASD032_father	1121	2265	8223	0.476	2285	21	10543	TRUE	TRUE	FALSE	0.02362
ASD033_child	ASD033_mother	1076	2241	8214	0.471	2207	18	10520	TRUE	TRUE	FALSE	0.02877
ASD033_child	ASD033_father	1109	2241	8191	0.474	2283	23	10520	TRUE	TRUE	FALSE	0.02566
ASD034_child	ASD034_mother	2208	2228	10614	0.994	2221	0	10638	TRUE	TRUE	FALSE	-0.4941
ASD034_child	ASD034_father	814	2228	7316	-0.02	2296	425	10638	TRUE	FALSE	TRUE	0.5162
ASD035_child	ASD035_mother	1051	2203	8220	0.46	2225	19	10553	TRUE	TRUE	FALSE	0.04017
ASD035_child	ASD035_father	1138	2203	8333	0.509	2294	8	10553	TRUE	TRUE	FALSE	-0.009305
ASD036_child	ASD036_mother	1151	2255	8338	0.497	2273	15	10571	TRUE	TRUE	FALSE	0.002882
ASD036_child	ASD036_father	1143	2255	8328	0.491	2269	18	10571	TRUE	TRUE	FALSE	0.009091
ASD037_child	ASD037_mother	1167	2304	8187	0.495	2362	13	10525	TRUE	TRUE	FALSE	0.004774
ASD037_child	ASD037_father	1108	2304	8270	0.497	2165	16	10525	TRUE	TRUE	FALSE	0.003002
ASD038_child	ASD038_mother	1043	2174	8284	0.467	2208	14	10593	TRUE	TRUE	FALSE	0.03312
ASD038_child	ASD038_father	1104	2174	8320	0.5	2303	9	10593	TRUE	TRUE	FALSE	0.00046
ASD039_child	ASD039_mother	1109	2244	8234	0.476	2285	21	10560	TRUE	TRUE	FALSE	0.02451
ASD039_child	ASD039_father	1092	2244	8238	0.475	2251	13	10560	TRUE	TRUE	FALSE	0.02496
ASD040_child	ASD040_mother	1086	2236	8267	0.484	2194	12	10532	TRUE	TRUE	FALSE	0.01595
ASD040_child	ASD040_father	1126	2236	8192	0.498	2353	6	10532	TRUE	TRUE	FALSE	0.001789
ASD041_child	ASD041_mother	1160	2372	8141	0.488	2335	10	10529	TRUE	TRUE	FALSE	0.01178
ASD041_child	ASD041_father	1179	2372	8221	0.499	2290	18	10529	TRUE	TRUE	FALSE	0.0008734
ASD042_child	ASD042_mother	1078	2224	8214	0.474	2211	15	10498	TRUE	TRUE	FALSE	0.02601
ASD042_child	ASD042_father	1148	2224	8158	0.502	2410	16	10498	TRUE	TRUE	FALSE	-0.001799

sample_a	sample_b	shared_hets	hets_a	ibs2	rel	hets_b	ibs0	n	pedigree_parents	predicted_parents	parent_error	rel difference
ASD043_child	ASD043_mother	1992	3573	7537	0.583	3378	12	10005	TRUE	TRUE	FALSE	-0.08259
ASD043_child	ASD043_father	1533	3573	7411	0.662	2279	12	10005	TRUE	TRUE	FALSE	-0.1621
ASD044_child	ASD044_mother	1188	2313	8161	0.497	2405	19	10513	TRUE	TRUE	FALSE	0.00281
ASD044_child	ASD044_father	1168	2313	8195	0.489	2330	19	10513	TRUE	TRUE	FALSE	0.01146
ASD045_child	ASD045_mother	1140	2299	8229	0.485	2310	12	10556	TRUE	TRUE	FALSE	0.01457
ASD045_child	ASD045_father	1208	2299	8305	0.517	2371	10	10556	TRUE	TRUE	FALSE	-0.01675
ASD046_child	ASD046_mother	1117	2296	8171	0.477	2327	11	10568	TRUE	TRUE	FALSE	0.02308
ASD046_child	ASD046_father	1092	2296	8095	0.464	2355	13	10568	TRUE	TRUE	FALSE	0.03571
ASD047_child	ASD047_mother	1100	2215	8171	0.477	2287	22	10482	TRUE	TRUE	FALSE	0.02325
ASD047_child	ASD047_father	1070	2215	8150	0.466	2245	19	10482	TRUE	TRUE	FALSE	0.03409
ASD048_child	ASD048_mother	1076	2231	8223	0.477	2232	6	10531	TRUE	TRUE	FALSE	0.02308
ASD048_child	ASD048_father	1115	2231	8258	0.485	2258	16	10531	TRUE	TRUE	FALSE	0.01457
ASD049_child	ASD049_mother	1071	2222	8174	0.474	2204	13	10467	TRUE	TRUE	FALSE	0.02586
ASD049_child	ASD049_father	1079	2222	8130	0.469	2261	18	10467	TRUE	TRUE	FALSE	0.0306
ASD050_child	ASD050_mother	1076	2222	8123	0.472	2264	14	10467	TRUE	TRUE	FALSE	0.02835
ASD050_child	ASD050_father	1021	2222	8169	0.466	2113	18	10467	TRUE	TRUE	FALSE	0.03384
ASD051_child	ASD051_mother	1131	2229	8224	0.491	2296	18	10496	TRUE	TRUE	FALSE	0.008748
ASD051_child	ASD051_father	1154	2229	8304	0.504	2275	15	10496	TRUE	TRUE	FALSE	-0.004262
ASD052_child	ASD052_mother	1134	2277	8268	0.485	2303	15	10592	TRUE	TRUE	FALSE	0.01515
ASD052_child	ASD052_father	1112	2277	8267	0.476	2273	15	10592	TRUE	TRUE	FALSE	0.02398
ASD053_child	ASD053_mother	1107	2266	8175	0.48	2276	10	10496	TRUE	TRUE	FALSE	0.0203
ASD053_child	ASD053_father	1088	2266	8127	0.472	2281	9	10496	TRUE	TRUE	FALSE	0.0278
ASD054_child	ASD054_mother	1125	2328	8182	0.485	2258	15	10525	TRUE	TRUE	FALSE	0.01506
ASD054_child	ASD054_father	1200	2328	8254	0.506	2340	11	10525	TRUE	TRUE	FALSE	-0.006014
ASD056_child	ASD056_mother	1039	2182	8147	0.459	2193	19	10455	TRUE	TRUE	FALSE	0.04125
ASD056_child	ASD056_father	1059	2182	8186	0.467	2185	20	10455	TRUE	TRUE	FALSE	0.033
ASD057_child	ASD057_mother	1101	2183	8238	0.487	2283	19	10517	TRUE	TRUE	FALSE	0.01306

sample_a	sample_b	shared_hets	hets_a	ibs2	rel	hets_b	ibs0	n	pedigree_parents	predicted_parents	parent_error	rel difference
ASD057_child	ASD057_father	1049	2183	8196	0.467	2221	15	10517	TRUE	TRUE	FALSE	0.03321
ASD058_child	ASD058_mother	1122	2276	8194	0.487	2295	7	10524	TRUE	TRUE	FALSE	0.01318
ASD058_child	ASD058_father	1174	2276	8300	0.505	2295	12	10524	TRUE	TRUE	FALSE	-0.005272
ASD059_child	ASD059_mother	1143	2294	8213	0.486	2312	14	10542	TRUE	TRUE	FALSE	0.01395
ASD059_child	ASD059_father	1143	2294	8228	0.485	2304	15	10542	TRUE	TRUE	FALSE	0.01482
ASD060_child	ASD060_mother	1134	2308	8305	0.495	2259	8	10612	TRUE	TRUE	FALSE	0.005091
ASD060_child	ASD060_father	1147	2308	8291	0.493	2299	7	10612	TRUE	TRUE	FALSE	0.007177
ASD061_child	ASD061_mother	1177	2392	8197	0.492	2317	18	10569	TRUE	TRUE	FALSE	0.007553
ASD061_child	ASD061_father	1226	2392	8207	0.503	2421	11	10569	TRUE	TRUE	FALSE	-0.003344
ASD062_child	ASD062_mother	1238	2383	8257	0.51	2437	11	10602	TRUE	TRUE	FALSE	-0.01028
ASD062_child	ASD062_father	1163	2383	8232	0.497	2311	7	10602	TRUE	TRUE	FALSE	0.002813
ASD063_child	ASD063_mother	1161	2351	8228	0.491	2330	8	10592	TRUE	TRUE	FALSE	0.008584
ASD063_child	ASD063_father	1174	2351	8276	0.502	2316	6	10592	TRUE	TRUE	FALSE	-0.001727
ASD064_child	ASD064_mother	1096	2252	8254	0.479	2241	11	10560	TRUE	TRUE	FALSE	0.02075
ASD064_child	ASD064_father	1152	2252	8290	0.506	2323	6	10560	TRUE	TRUE	FALSE	-0.006217
ASD065_child	ASD065_mother	1151	2321	8188	0.486	2338	11	10550	TRUE	TRUE	FALSE	0.01357
ASD065_child	ASD065_father	1122	2321	8207	0.483	2249	18	10550	TRUE	TRUE	FALSE	0.01712
ASD066_child	ASD066_mother	1134	2312	8191	0.474	2316	19	10559	TRUE	TRUE	FALSE	0.02595
ASD066_child	ASD066_father	1130	2312	8187	0.474	2313	17	10559	TRUE	TRUE	FALSE	0.02595
ASD067_child	ASD067_mother	1091	2290	8154	0.464	2305	14	10574	TRUE	TRUE	FALSE	0.03581
ASD067_child	ASD067_father	1101	2290	8199	0.476	2276	9	10574	TRUE	TRUE	FALSE	0.02417
ASD068_child	ASD068_mother	1117	2270	8278	0.482	2296	11	10614	TRUE	TRUE	FALSE	0.01762
ASD068_child	ASD068_father	1169	2270	8348	0.504	2326	13	10614	TRUE	TRUE	FALSE	-0.003524
ASD069_child	ASD069_mother	1128	2265	8292	0.485	2325	15	10633	TRUE	TRUE	FALSE	0.01523
ASD069_child	ASD069_father	1148	2265	8341	0.494	2313	15	10633	TRUE	TRUE	FALSE	0.006402
ASD070_child	ASD070_mother	1204	2430	8195	0.502	2354	11	10572	TRUE	TRUE	FALSE	-0.002124
ASD070_child	ASD070_father	1198	2430	8256	0.52	2281	6	10572	TRUE	TRUE	FALSE	-0.01995

sample_a	sample_b	shared_hets	hets_a	ibs2	rel	hets_b	ibs0	n	pedigree_parents	predicted_parents	parent_error	rel difference
ASD071_child	ASD071_mother	1187	2306	8319	0.503	2350	14	10612	TRUE	TRUE	FALSE	-0.002602
ASD071_child	ASD071_father	1139	2306	8257	0.484	2321	12	10612	TRUE	TRUE	FALSE	0.01648
ASD072_child	ASD072_mother	1161	2251	8348	0.503	2275	14	10557	TRUE	TRUE	FALSE	-0.003332
ASD072_child	ASD072_father	1072	2251	8190	0.455	2242	26	10557	TRUE	TRUE	FALSE	0.04505
ASD073_child	ASD073_mother	1219	2355	8294	0.511	2364	8	10589	TRUE	TRUE	FALSE	-0.01083
ASD073_child	ASD073_father	1150	2355	8300	0.509	2234	7	10589	TRUE	TRUE	FALSE	-0.008505
ASD074_child	ASD074_mother	1150	2319	8225	0.485	2373	13	10621	TRUE	TRUE	FALSE	0.01531
ASD074_child	ASD074_father	1093	2319	8310	0.494	2174	10	10621	TRUE	TRUE	FALSE	0.00644
ASD075_child	ASD075_mother	1176	2348	8274	0.497	2356	5	10625	TRUE	TRUE	FALSE	0.003407
ASD075_child	ASD075_father	1139	2348	8282	0.496	2265	8	10625	TRUE	TRUE	FALSE	0.004194
ASD076_child	ASD076_mother	1167	2315	8263	0.497	2373	8	10615	TRUE	TRUE	FALSE	0.002808
ASD076_child	ASD076_father	1127	2315	8246	0.48	2308	10	10615	TRUE	TRUE	FALSE	0.02036
ASD077_child	ASD077_mother	1127	2299	8258	0.475	2325	17	10636	TRUE	TRUE	FALSE	0.02458
ASD077_child	ASD077_father	1160	2299	8344	0.496	2305	10	10636	TRUE	TRUE	FALSE	0.004132
ASD078_child	ASD078_mother	1202	2359	8220	0.502	2402	9	10589	TRUE	TRUE	FALSE	-0.001908
ASD078_child	ASD078_father	1145	2359	8216	0.481	2281	24	10589	TRUE	TRUE	FALSE	0.01907
ASD079_child	ASD079_mother	1149	2281	8316	0.498	2277	8	10582	TRUE	TRUE	FALSE	0.002415
ASD079_child	ASD079_father	1041	2281	8251	0.478	2126	12	10582	TRUE	TRUE	FALSE	0.02164
ASD080_child	ASD080_mother	1129	2317	8164	0.486	2221	25	10458	TRUE	TRUE	FALSE	0.01418
ASD080_child	ASD080_father	814	2317	7008	-0.05	2288	467	10458	TRUE	FALSE	TRUE	0.5524
ASD081_child	ASD081_mother	1044	2313	8226	0.476	2155	9	10605	TRUE	TRUE	FALSE	0.0239
ASD081_child	ASD081_father	1161	2313	8298	0.494	2308	11	10605	TRUE	TRUE	FALSE	0.006499
ASD082_child	ASD082_mother	1078	2208	8130	0.468	2269	22	10467	TRUE	TRUE	FALSE	0.0317
ASD082_child	ASD082_father	1098	2208	8180	0.485	2263	14	10467	TRUE	TRUE	FALSE	0.0154
ASD083_child	ASD083_mother	1074	2179	8185	0.47	2186	25	10406	TRUE	TRUE	FALSE	0.03006
ASD083_child	ASD083_father	1103	2179	8136	0.493	2297	14	10406	TRUE	TRUE	FALSE	0.006654
ASD084_child	ASD084_mother	1085	2243	8207	0.475	2205	19	10504	TRUE	TRUE	FALSE	0.02517

sample_a	sample_b	shared_hets	hets_a	ibs2	rel	hets_b	ibs0	n	pedigree_parents	predicted_parents	parent_error	rel difference
ASD084_child	ASD084_father	1059	2243	8127	0.459	2246	15	10504	TRUE	TRUE	FALSE	0.04124
ASD085_child	ASD085_mother	1131	2245	8217	0.49	2325	15	10541	TRUE	TRUE	FALSE	0.009577
ASD085_child	ASD085_father	1092	2245	8187	0.479	2295	8	10541	TRUE	TRUE	FALSE	0.02071
ASD086_child	ASD086_mother	1081	2221	8112	0.462	2158	42	10385	TRUE	TRUE	FALSE	0.038
ASD086_child	ASD086_father	1106	2221	8049	0.471	2303	30	10385	TRUE	TRUE	FALSE	0.02904
ASD087_child	ASD087_mother	1077	2241	8200	0.462	2235	22	10535	TRUE	TRUE	FALSE	0.03781
ASD087_child	ASD087_father	1080	2241	8200	0.469	2245	14	10535	TRUE	TRUE	FALSE	0.03057
ASD088_child	ASD088_mother	1126	2243	8262	0.49	2265	13	10530	TRUE	TRUE	FALSE	0.009585
ASD088_child	ASD088_father	1065	2243	8200	0.465	2210	19	10530	TRUE	TRUE	FALSE	0.03529
ASD089_child	ASD089_mother	1101	2208	8276	0.476	2216	25	10511	TRUE	TRUE	FALSE	0.024
ASD089_child	ASD089_father	1080	2208	8231	0.467	2210	24	10511	TRUE	TRUE	FALSE	0.03261
ASD090_child	ASD090_mother	1086	2209	8217	0.468	2194	30	10470	TRUE	TRUE	FALSE	0.03236
ASD090_child	ASD090_father	1063	2209	8146	0.459	2230	25	10470	TRUE	TRUE	FALSE	0.04142
ASD091_child	ASD091_mother	1101	2216	8223	0.485	2274	13	10520	TRUE	TRUE	FALSE	0.01489
ASD091_child	ASD091_father	731	2216	7049	-0.1	2242	478	10520	TRUE	FALSE	TRUE	0.6015
ASD092_child	ASD092_mother	1107	2253	8231	0.479	2232	19	10530	TRUE	TRUE	FALSE	0.02106
ASD092_child	ASD092_father	1120	2253	8198	0.484	2315	15	10530	TRUE	TRUE	FALSE	0.0162
ASD093_child	ASD093_mother	1129	2370	8103	0.473	2254	31	10484	TRUE	TRUE	FALSE	0.02662
ASD093_child	ASD093_father	1179	2370	8146	0.499	2324	10	10484	TRUE	TRUE	FALSE	0.001291
ASD094_child	ASD094_mother	1120	2261	8271	0.501	2183	13	10486	TRUE	TRUE	FALSE	-0.001145
ASD094_child	ASD094_father	1089	2261	8110	0.452	2264	34	10486	TRUE	TRUE	FALSE	0.04843
ASD095_child	ASD095_mother	1118	2313	8173	0.483	2288	7	10540	TRUE	TRUE	FALSE	0.01748
ASD095_child	ASD095_father	1091	2313	8201	0.486	2195	12	10540	TRUE	TRUE	FALSE	0.0139
ASD096_child	ASD096_mother	1123	2308	8321	0.491	2232	14	10623	TRUE	TRUE	FALSE	0.009409
ASD096_child	ASD096_father	1184	2308	8311	0.502	2357	13	10623	TRUE	TRUE	FALSE	-0.001733
ASD097_child	ASD097_mother	1135	2263	8303	0.484	2287	20	10600	TRUE	TRUE	FALSE	0.01613
ASD097_child	ASD097_father	1099	2263	8285	0.476	2245	15	10600	TRUE	TRUE	FALSE	0.02383

sample_a	sample_b	shared_hets	hets_a	ibs2	rel	hets_b	ibs0	n	pedigree_parents	predicted_parents	parent_error	rel difference
ASD098_child	ASD098_mother	1130	2210	8271	0.492	2350	21	10577	TRUE	TRUE	FALSE	0.007692
ASD098_child	ASD098_father	1057	2210	8227	0.462	2235	18	10577	TRUE	TRUE	FALSE	0.03801
ASD099_child	ASD099_mother	1092	2213	8262	0.474	2263	22	10562	TRUE	TRUE	FALSE	0.02643
ASD099_child	ASD099_father	1113	2213	8262	0.48	2303	25	10562	TRUE	TRUE	FALSE	0.01966
ASD100_child	ASD100_mother	1151	2300	8340	0.497	2274	11	10615	TRUE	TRUE	FALSE	0.003518
ASD100_child	ASD100_father	842	2300	7312	0.022	2295	396	10615	TRUE	FALSE	TRUE	0.4782
ASD101_child	ASD101_mother	862	2323	7236	-0.01	2335	437	10602	TRUE	FALSE	TRUE	0.5052
ASD101_child	ASD101_father	815	2323	7269	-0.01	2231	417	10602	TRUE	FALSE	TRUE	0.5085
ASD102_child	ASD102_mother	1125	2336	8247	0.487	2267	10	10601	TRUE	TRUE	FALSE	0.01257
ASD102_child	ASD102_father	1147	2336	8274	0.495	2279	10	10601	TRUE	TRUE	FALSE	0.005485
ASD103_child	ASD103_mother	1115	2274	8245	0.482	2259	13	10551	TRUE	TRUE	FALSE	0.01793
ASD103_child	ASD103_father	1101	2274	8244	0.478	2234	17	10551	TRUE	TRUE	FALSE	0.02238

Supplementary Table S4. Phenotype-genotype analysis

#Proband ASD005

This male proband was delivered via C-section with a body weight of 3.8 kg. He was the 4th child of healthy parents with no family history of genetic disease. The mother was 42 years old (y/o) when he was delivered. He began to walk at 2 y/o and was reported to have a speech delay. He could speak 2-3 single words at the age of 3. He presented typical syndromes of ASD, such as nonsense talk, hyperactivity, toe walking, spinning, repetitive behaviours (door closing, light switching), poor social interaction and sensory processing disorders (smelling and tasting food). At 45 m/o, his DSM-V was at level 3 with a CARS score of 49.5, his language was equal to that at 12 months old (m/o) and his understanding was equal to that at 8.5 m/o (Supplementary Table S1). This male proband harboured a missense X-linked variant that was inherited from his mother, TBX22:c.452G>T [p.Arg151Leu] (Table 1). TBX22 acts as a transcriptional repressor and is expressed in early development[1]. Several *TBX22* mutations have been identified to cause cleft palate and ankyloglossia[1,2] (Table 3). However, this proband did not show cleft palate or ankyloglossia. All *in silico* tools predicted that this variant was damaging (Table 2). This gene has not been previously reported in any autism database (Table 3).

#Proband ASD006

This female proband was delivered via C-section (3.4 kg at birth). She began to walk and to babble at 13 m/o. Soon after, the parents reported that her language and intellectual abilities started to regress by 18 m/o. After 2 y/o, she developed jargon speech, lack of eye contact and hypersensitive hearing. She did not chew food and was interested in drawing and smart phones. PET/CT examination showed hypometabolism in the temporal lobe and bilateral hippocampus and decreased FDG metabolism at moderate levels in the prefrontal region and the parietal lobe (Supplementary Table S1). We detected a missense *de novo* variant (IGF1*:*c.251G>A [p. Arg84Aln]) from this proband (Table 1). This variant was predicted to be damaging (Table 2). *IGF1* encodes insulin-like growth factor 1, which is involved in the regulation of [1-14C]-2-deoxy-D-glucose transport and glycogen synthesis in osteoblasts, stimulation of glucose transport in bone-derived osteoblastic cells[3], synapse maturation[4], integrin binding[5,6] and activation of other growth factors[7] (Table 3, Fig. 2). This gene has not been recorded in the autism databases (Table 3). However, *IGF1* mutations have been identified to be associated with hypoglycaemia and postnatal growth disorders[8].

#Proband ASD032

This male proband was the 1st child and delivered via vaginal birth (3.8 kg at birth). He started walking at 13 m/o. The parents reported that he experienced seizures when he was 4 y/o and often had constipation (Supplementary Table S1). His presentations included hyperactivity and poor social interaction, and he was unresponsive when called. He was diagnosed with ASD at 20 m/o. By the age of 7, he could speak some single words, retaining attention deficits and other autistic symptoms. He carried a *de novo* frameshift variant (MBD5:c.14_15delAA [p.Lys5fs]). MutationTaster and SnpEff indicated that this genetic predisposition was damaging (Table 2). *MBD5* encodes a methyl-CpG-binding domain protein that is involved in numerous cellular processes, such as cell division, growth and differentiation[9,10]. Mutations in this gene have been detected in patients with unexplained ID[11], EP and ASD[12-14] and recorded in the SFARI and AutDB databases (Table 3).

#Proband ASD035

This male proband was delivered by forceps. He began to walk at 17 m/o and to babble at 24 m/o. He showed a delay in speech and was later diagnosed with ASD at the age of 3. By the enrolment time in this study, he was diagnosed with ASD at a severe level (DSM-V level 3, ADOS score: 20/12) (Supplementary Table S1). He could speak 2-3 single words when required and showed poor cognition, lack of eye contact, poor social interaction and hyperactivity. He often smelled and licked food before eating and mouthed objects. His MRI showed abnormalities in which the left lateral ventricles were larger than the right ventricles. This proband harboured an X-linked variant ATRX:c.1972C>T [p.Arg658Cys] that was predicted to be damaging. A rare *ATRX* mutation has been identified in an individual with ASD[15], and *ATRX* has been recorded in the SFARI, AutDB and AutismKB databases (Table 3).

#Proband ASD038

This male proband was delivered via C-section (2.6 kg at birth). He was reported to skip some milestones, including rolling and clawing. He showed ID and motor delay in early childhood and was diagnosed with ASD at the age of 2 with typical autistic symptoms (lack of eye contact, hyperactivity and nonverbal). He began to walk at 2 y/o and experienced EP. At 48 m/o, his language was equal to an 8 m/o, and the developmental quotient (DQ) was 20% (Supplementary Table S1). Brain MRI showed no abnormalities. We detected a *de novo* stop gained variant DYRK1A:c.601C>T [p.Gln201*] from this proband (Table 1). *DYRK1A*

encodes a protein member of the dual-specificity tyrosine phosphorylation-regulated kinase family and participates in various cellular processes, such as protein modifications, DNA damage responses, signalling transductions and circadian rhythms[16]. *DYRK1A* is highly conserved and located on chromosome 21, which was shown to cause the majority of phenotypic features in Down syndrome, such as mental retardation (MR), microcephaly and dysmorphic features[17] (Table 3). Recently, another study identified several *DYRK1A* mutations in individuals with ASD[18]. In our study, this detected variant caused a stop codon that was predicted to be damaging (Table 2). *DYRK1A* was identified as an ASD risk gene[19] and categorized as a syndromic ASD gene in SFARI (Table 3).

# Proband ASD046

This male proband was vaginally delivered and was 3.2 kg at birth. At the age of 11 m/o, he was diagnosed with ID. He began to babble at 18-20 m/o and showed language regression after that. At the age of 2 y/o, he was diagnosed with ASD. He was able to walk at age 3. By recruitment time, he exhibited delays in motor and intellectual activity, hyperactivity, self-talk, lack of eye contact, poor social interaction, attention deficit, aggressive sensory processing disorders, and good memory on visualization (DSM-V: level 3, ADOS score: 21/12 and CARS score: 46). Not surprisingly, at 12 y/o, his fine motor and gross motor were merely equal to a 6 y/o (Supplementary Table S1). He was also diagnosed with CP, although brain MRI showed no abnormalities. This male proband carried two LoF variants: a *de novo* variant GRIN2B:c.2208dupG [p.Asn737fs] and an X-linked variant AGTR2:c.757C>T [p.Gln253*] (Table 1). Noticeably, the X-linked variant AGTR2:c.757C>T [p.Gln253*] was predicted as deleterious by CADD and MutationTaster (Table 2). *GRIN2B* encodes a subunit of the N-methyl D aspartate (NMDA) receptor ion channel. The NMDA receptor is a glutamate-activated ion channel permeable to Na^+, K^+, Ca^{2+}, which acts as an agonist binding site for glutamate and is found at excitatory synapses throughout the brain[20]. The early expression of *GRIN2B* in the brain during prenatal development suggests its role in brain development, circuit formation, synaptic plasticity, and cellular migration and differentiation. Mutations within this gene are associated with neurodevelopmental disorders, including ASD, ADHD, EP and schizophrenia[21] (Table 3). Meanwhile, *AGTR2,* located on the X chromosome, encodes a protein member of the G-protein coupled receptor 1 family, which functions as a receptor for angiotensin II. This receptor mediates programmed cell death, and this apoptotic function may play an important role in developmental biology and pathophysiology[22]. *AGTR2* mutations

have been found to be associated with X-linked cognitive disability[23] and recorded in SFARI, AutDB and AutismKB (Table 3).

#Proband ASD056

This male proband was the 2nd child and delivered vaginally and was 3.4 kg at birth. He began to walk at the age of 17-18 m/o and to babble at 24 m/o. He was reportedly regressed in his language. By the age of 2, he could speak a few single words and did not show any progression. At age 3, he was diagnosed with ASD (Supplementary Table S1). His presentations were objects biting, spinning and unresponsive when the parents called. At the age of 4 y/o, he could speak some simple sentences and retained autistic behaviours such as hand waving, nail biting, hyperactivity, nonsense talking, poor social interaction and smell sensitivity. Brain MRI showed no abnormality detected (Supplementary Table S1). This proband harboured a *de novo* deletion variant: ENPP1:c.1081_1083delAAA [p.Lys361del]. MutationTaster predicted that this variant was damaging (Table 2). A search against the autism database showed that this gene has not been previously reported to be linked to ASD or other neurological disorders (Table 3) but was associated with insulin resistance[24], 'idiopathic' infantile arterial calcification[25,26] and Cole disease[27].

#Proband ASD057

This male proband was delivered via C-section (2.5 kg at birth). He was able to walk at 12 m/o but exhibited speech delay. He was diagnosed with ASD at 3 y/o. His presentations included poor social interaction, nonverbal, hyperactivity, repetitive behaviours, lack of eye contact, toe walking, attention deficit and spinning. He was interested in door closing and was not able to differentiate colours. He carried an X-linked variant in the *DMD* gene: DMD:c.1138C>T [p.His380Tyr] (Table 1). *DMD* encodes dystrophin, which plays a role in bridging the inner cytoskeleton and the extracellular matrix. Extensive deletions, duplications and point mutations in the *DMD* gene have been reported to cause Becker muscular dystrophy[28], Duchenne muscular dystrophy[29] or cardiomyopathy[30,31]. The proband showed gross motor skills that were equal to his age. Nevertheless, *DMD* has been identified as an ASD risk gene as recorded in the autism database (Table 3). Our prediction tools showed that this missense variant was damaging (Table 2).

#Proband ASD059

This proband was the 2nd child and delivered via C-section (3 kg at birth). The parents reported that he showed normal motor skills but speech delay. He was diagnosed with ASD at 22 m/o (Supplementary Table S1). His clinical expressions included hyperactivity, sensory disorders, lack of eye contact, nonverbal, ID and toe walking. At the age of 7, he could speak 5-6 single words but remained isolated and had poor social interaction. He did not like the colour red and sour foods. His brain MRI showed no abnormality. Interestingly, like proband ASD057 as shown above, this male proband also carried a missense X-linked variant in the *DMD* gene. MutationTaster predicted that variant DMD:c.2877A>C [p.Glu959Asp] was damaging (Table 2).

#Proband ASD063

This male proband was delivered via C-section (4.1 kg at birth). He began to walk at the age of 15 m/o and soon exhibited speech delay and toe walking and was strongly interested in TV advertisements, but no seizures were reported. He was diagnosed with ASD at the age of 3 (Supplementary Table S1) and then participated in an educational program for children with ASD but did not show significant progress. At 5 y/o, he could speak 2-3 single words when requested. He showed autistic symptoms (hyperactivity, spinning, lack of eye contact, attention deficit, smelling and licking food before eating and hypersensitive hearing). PET/CT showed a decrease in FDG metabolism in the hippocampus region and in the frontal lobe, but brain MRI indicated no abnormality. We found a *de novo* missense variant in the *CHD8* gene (c.3575T>C [p.Ile1192Thr]) from this proband (Tables 1, 2). *CHD8* encodes chromodomain-helicase-DNA binding protein 8, which is involved in epigenetic remodelling, promotion of cell proliferation and regulation of RNA synthesis[32,33]. Allelic *CHD8* mutations have been reported to associate with ASD[18,34,35], and this gene has been listed in the autism databases (Table 3).

# Proband ASD068

This female proband was the 3rd child of healthy parents. Her siblings were healthy. She was vaginally delivered (4.2 kg at birth) and began to walk by 12 m/o. The parents reported that she had seizures at 24 m/o and expressed speech delays. She was nonverbal and showed hyperactivity, lack of eye contact and sensory disorders. She also showed some symptoms of EP. However, her brain MRI showed no abnormalities (Supplementary Table S1). This girl carried a *de novo* frameshift variant, SCN2A:c.232delC [p.Leu78fs], which was predicted to be damaging (Table 2). *SCN2A* has been considered an ASD risk gene[19] and is reported in the

autism databases (Table 3). *SCN2A* encodes the sodium voltage-gated channel alpha subunit 2 protein, which is involved in the generation and propagation of action potentials[36]. Allelic *SCN2A* variants have been found to be associated with seizure[37], early infantile epileptic encephalopathy[38] and ASD[35,39]. Hence, the genotype-phenotype of this female proband was consistent with previous studies suggesting that *SCN2A* was a universal gene for ASD.

#Proband ASD076

This male proband was the 1st child and delivered via C-section (3.7 kg at birth). He began to walk at the age of 14 m/o. As reported, his language regressed after 12 m/o (before that, he could babble some words) (Supplementary Table S1). At the age of 2, he was highly interested in TV or screens and was interested in spinning. At 5 y/o, he could not speak a single word, loved to run around and frequently laughed and cried. He could not differentiate strangers from his relatives and did not play with friends. He was hyperactive (often jumping from high positions) and often screamed. He has been progressively developing more severe symptoms. PET/CT showed decreased FDG uptake in the hippocampus and in the frontal lobe (Supplementary Table S1). We found two X-linked variants from this proband: SYP:c.251C>G [p.Ala84Gly] and LAS1L:c.1797_1805delTGATGAAGA [p.Asp599_Glu601del] (Table 1). Notably, neither the *SYP* nor the *LAS1L* gene has been previously reported to be linked to ASD (Table 3). *SYP* encodes synaptophysin, which plays a role in synaptic vesicle trafficking[40]. Mutations in the *SYP* gene have been found to associate with several X-linked forms of MR[41]. A dozen variants in this gene have been submitted in ClinVar ranging from benign to pathogenic, but most cases are classified as pathogenic. Our reported variant here was novel with no record in dbSNP (Table 1). Meanwhile, *LAS1L* (LAS1 like, ribosome biogenesis factor) l encodes a subunit of a nucleolar protein complex that is involved in the processing of 47S pre-rRNA to 28S and 5.8S rRNA and is required for the synthesis of the 60S ribosomal subunit[42,43]. *LAS1L* mutations have been found to associate with Wilson-Turner X-linked MR[44,45].

#Proband ASD083

This male proband was healthy at birth and could reach some milestones during his early infancy. He was delivered by C-section with a birth weight of 3.2 kg. He began to babble and to walk at 9 m/o. He did not experience seizures. However, after that, he developed autistic symptoms (speech delay, spinning, hyperactivity, hypersensitive hearing, eating disorder, nonverbal, lack of eye contact and toe walking). He was diagnosed with ASD at 26 m/o (ADOS

score of 20/12, CARS score of 46) (Supplementary Table S1). He carried an X-linked variant in the *OFD1* gene (c.2209A>G [p.Thr737Ala]) which was predicted to be damaging (Table 1, 2). *OFD1* encodes the centrosomal protein required for primary cilia formation[46,47]. *OFD1* mutations are associated with multiple human genetic disorders known as ciliopathies, which include oral-facial-digital syndrome type I (OFDS1) and Simpson-Golabi-Behmel syndrome type 2 and Joubert syndrome and retinitis pigmentosa (Table 3). In addition, OFD1 plays a crucial role in forebrain development and in the control of dorsoventral patterning and early corticogenesis during embryonic development[48]. This gene has been previously reported in patients with ASD and identified as a risk factor for ASD[49].

#Proband ASD086

This male proband was delivered via C-section (4 kg at birth). He was able to walk at 12 m/o. His clinical presentations included speech delay, unresponsiveness, poor social interaction, nonverbal, toe walking, fear of loud sounds, unreasonable laughing, lack of eye contact and hyperactivity. He only drank milk and ate porridge. He was first diagnosed with ASD at 17 m/o (Supplementary Table S1). This proband carried an X-linked missense variant (CHM:c.866T>C [p.Met289Thr]) (Table 1). *CHM* has not been previously reported in the autism databases (Table 3). Previous studies have detected *CHM* mutations in patients with clinical characterizations of slowly progressive degeneration of the choroid, photoreceptors and retinal pigment epithelium[50,51]. SIFT and MutationTaster predicted that this variant was damaging, but PolyPhen-2 predicted that it was benign (Table 2).

#Proband ASD097

This male proband was the 2nd child. His sibling was healthy as reported. He was vaginally delivered with a birth weight of 3.2 kg. He began to walk at the age of 2, exhibited speech delay, was interested in TV advertisement programs, showed toe walking and spinning, was nonverbal, displayed unreasonable laughing and crying, had poor social interaction, and was hyperactive, but no seizures were reported. His brain MRI indicated no abnormality detected. He was diagnosed with ASD at 24 m/o (Supplementary Table S1). This proband harboured a *de novo* missense variant SLC16A7:c.260C>T [p.Pro87Leu] (Table 1). *SLC16A7* encodes monocarboxylate transporter 2, which plays a role in the transport of pyruvate and lactate across cellular membranes in mammalian cells[52]. *SLC16A* is categorized as a hypothesized gene to ASD in the SFARI database (Table 3). A *de novo* CNV variant in the *SLC16A7* gene has been detected in a male patient with ASD and hyperactivity[53].

#Proband ASD099

This male proband was vaginally delivered at 38 weeks of gestation with a birth weight of 3.7 kg. He began to babble at 12 m/o and to walk at 14 m/o. He showed sleep deprivation (often sleeping at 2-3 AM), was nonverbal, was unresponsive when called, and showed toe walking, poor social interaction, and eating disorder. He was diagnosed with ASD at 24 m/o (Supplementary Table S1). When he was 5 y/o, brain MRI diagnosis suggested Chiari type I disorder. We found an X-linked variant in the *PLXNA3* gene from this proband (c.2113C>T [p.Arg705Trp]) (Table 1). *PLXNA3* encodes the plexin 3 protein, which is involved in regulating neuronal axon guidance. *PLXNA3* mutations may lead to the malfunction of plexin signalling, leading to neurological disorders and cancers[54]. A *de novo* splice site *PLXNA3* variant has been detected in a patient with ASD[55]. Other rare *PLXNA3* variants have been reported in a Chinese cohort with ASD[49] (Table 3).

References

1 Braybrook, C. *et al.* Craniofacial expression of human and murine *TBX22* correlates with the cleft palate and ankyloglossia phenotype observed in CPX patients. *Hum Mol Genet* **11**, 2793-2804, doi:10.1093/hmg/11.22.2793 (2002).

2 Marçano, A. C. B. *et al. TBX22* mutations are a frequent cause of cleft palate. *J Med Genet* **41**, 68-74, doi:10.1136/jmg.2003.010868 (2004).

3 Zoidis, E., Ghirlanda-Keller, C. & Schmid, C. Stimulation of glucose transport in osteoblastic cells by parathyroid hormone and insulin-like growth factor I. *Mol Cell Biochem* **348**, 33-42, doi:10.1007/s11010-010-0634-z (2011).

4 Shcheglovitov, A. *et al.* SHANK3 and IGF1 restore synaptic deficits in neurons from 22q13 deletion syndrome patients. *Nature* **503**, 267-271, doi:10.1038/nature12618 (2013).

5 Saegusa, J. *et al.* The direct binding of insulin-like growth factor-1 (IGF-1) to integrin alphavbeta3 is involved in IGF-1 signaling. *J Biol Chem* **284**, 24106-24114, doi:10.1074/jbc.M109.013201 (2009).

6 Fujita, M. *et al.* Cross-talk between integrin *α6β4* and insulin-like growth factor-1 receptor (IGF1R) through direct *α6β4* binding to IGF1 and subsequent *α6β4*-IGF1-IGF1R ternary complex formation in anchorage-independent conditions. *J Biol Chem* **287**, 12491-12500, doi:10.1074/jbc.M111.304170 (2012).

7 Fujita, M., Takada, Y. K. & Takada, Y. Insulin-like growth factor (IGF) signaling requires αvβ3-IGF1-IGF type 1 receptor (IGF1R) ternary complex formation in anchorage independence, and the complex formation does not require IGF1R and Src activation. *J Biol Chem* **288**, 3059-3069, doi:10.1074/jbc.M112.412536 (2013).

8 Solomon-Zemler, R. *et al.* A novel heterozygous IGF-1 receptor mutation associated with hypoglycemia. *Endocrine Connections* **6**, 395-403, doi:10.1530/EC-17-0038 (2017).

9 Jung, B. P. *et al.* The expression of methyl CpG binding factor MeCP2 correlates with cellular differentiation in the developing rat brain and in cultured cells. *J Neurobiol* **55**, 86-96, doi:10.1002/neu.10201 (2003).

10 Yano, A. *et al.* Interaction between methyl CpG-binding protein and ran GTPase during cell division in tobacco cultured cells. *Ann Bot* **98**, 1179-1187, doi:10.1093/aob/mcl211 (2006).

11 Wagenstaller, J. *et al.* Copy-number variations measured by single-nucleotide-polymorphism oligonucleotide arrays in patients with mental retardation. *Am J Hum Genet* **81**, 768-779, doi:10.1086/521274 (2007).

12 Hodge, J. C. *et al.* Disruption of *MBD5* contributes to a spectrum of psychopathology and neurodevelopmental abnormalities. *Mol Psychiatry* **19**, 368-379, doi:10.1038/mp.2013.42 (2014).

13 Mullegama, S. V., Alaimo, J. T., Chen, L. & Elsea, S. H. Phenotypic and molecular convergence of 2q23.1 deletion syndrome with other neurodevelopmental syndromes associated with autism spectrum disorder. *Int J Biol Sci* **16**, 7627-7643, doi:10.3390/ijms16047627 (2015).

14 Williams, S. R. *et al.* Haploinsufficiency of MBD5 associated with a syndrome involving microcephaly, intellectual disabilities, severe speech impairment, and seizures. *Eur J Hum Genet* **18**, 436-441, doi:10.1038/ejhg.2009.199 (2010).

15 Gong, X. *et al.* Analysis of X chromosome inactivation in autism spectrum disorders. *Am J Med Genet B Neuropsychiatr Genet* **147B**, 830-835, doi:10.1002/ajmg.b.30688 (2008).

16 Baek, K.-H. *et al.* Down's syndrome suppression of tumour growth and the role of the calcineurin inhibitor DSCR1. *Nature* **459**, 1126, doi:10.1038/nature08062 (2009).

17 van Bon, B. W. M. *et al.* Intragenic deletion in *DYRK1A* leads to mental retardation and primary microcephaly. *Clin Genet* **79**, 296-299, doi:10.1111/j.1399-0004.2010.01544.x (2011).

18 O'Roak, B. J. *et al.* Multiplex targeted sequencing identifies recurrently mutated genes in autism spectrum disorders. *Science* **338**, 1619, doi:10.1126/science.1227764 (2012).

19 De Rubeis, S. *et al.* Synaptic, transcriptional and chromatin genes disrupted in autism. *Nature* **515**, 209-215, doi:10.1038/nature13772 (2014).

20 Matta, J. A., Ashby, M. C., Sanz-Clemente, A., Roche, K. W. & Isaac, J. T. R. mGluR5 and NMDA receptors drive the experience- and activity-dependent NMDA receptor NR2B to NR2A subunit switch. *Neuron* **70**, 339-351, doi:https://doi.org/10.1016/j.neuron.2011.02.045 (2011).

21 Endele, S. *et al.* Mutations in *GRIN2A* and *GRIN2B* encoding regulatory subunits of NMDA receptors cause variable neurodevelopmental phenotypes. *Nat Genet* **42**, 1021, doi:10.1038/ng.677 (2010).

22 Yamada, T., Horiuchi, M. & Dzau, V. J. Angiotensin II type 2 receptor mediates programmed cell death. *Proc Natl Acad Sci USA* **93**, 156, doi:10.1073/pnas.93.1.156 (1996).

23 Ylisaukko-oja, T. *et al.* Identification of two *AGTR2* mutations in male patients with non-syndromic mental retardation. *Hum Genet* **114**, 211-213, doi:10.1007/s00439-003-1048-8 (2004).

24 Pizzuti, A. *et al.* A polymorphism (K121Q) of the human glycoprotein PC-1 gene coding region is strongly associated with insulin resistance. *Diabetes* **48**, 1881, doi:10.2337/diabetes.48.9.1881 (1999).

25 Rutsch, F. *et al.* Hypophosphatemia, hyperphosphaturia, and bisphosphonate treatment are associated with survival beyond infancy in generalized arterial calcification of infancy. *Circulation. Cardiovascular Genetics* **1**, 133-140, doi:10.1161/CIRCGENETICS.108.797704 (2008).

26 Rutsch, F. *et al.* Mutations in *ENPP1* are associated with 'idiopathic' infantile arterial calcification. *Nat Genet* **34**, 379, doi:10.1038/ng1221 (2003).

27 Eytan, O. *et al.* Cole disease results from mutations in *ENPP1*. *Am J Hum Genet* **93**, 752-757, doi:https://doi.org/10.1016/j.ajhg.2013.08.007 (2013).

28 Zellweger, H. & Hanson, J. W. Slowly progressive X-linked recessive muscular dystrophy (type IIIb): report of cases and review of the literature. *Arch Intern Med* **120**, 525-535, doi:10.1001/archinte.1967.00300040009002 (1967).

29 Tuffery-Giraud, S. *et al.* Genotype–phenotype analysis in 2,405 patients with a dystrophinopathy using the UMD–DMD database: a model of nationwide knowledgebase. *Hum Mutat* **30**, 934-945, doi:10.1002/humu.20976 (2009).

30 Muntoni, F. *et al.* Deletion of the dystrophin muscle-promoter region associated with X-Linked dilated cardiomyopathy. *N Engl J Med* **329**, 921-925, doi:10.1056/NEJM199309233291304 (1993).

31 Ortiz-Lopez, R., Li, H., Su, J., Goytia, V. & Towbin Jeffrey, A. Evidence for a dystrophin missense mutation as a cause of X-Linked dilated cardiomyopathy. *Circulation* **95**, 2434-2440, doi:10.1161/01.CIR.95.10.2434 (1997).

32 Ishihara, K., Oshimura, M. & Nakao, M. CTCF-dependent chromatin insulator is linked to epigenetic remodeling. *Mol Cell* **23**, 733-742, doi:https://doi.org/10.1016/j.molcel.2006.08.008 (2006).

33 Sakamoto, I. *et al.* A novel β-catenin-binding protein inhibits β-catenin-dependent Tcf activation and axis formation. *J Biol Chem* **275**, 32871-32878, doi:10.1074/jbc.M004089200 (2000).

34 Neale, B. M. *et al.* Patterns and rates of exonic de novo mutations in autism spectrum disorders. *Nature* **485**, 242, doi:10.1038/nature11011 (2012).

35 Sanders, S. J. *et al. De novo* mutations revealed by whole-exome sequencing are strongly associated with autism. *Nature* **485**, 237-241, doi:10.1038/nature10945 (2012).

36 Garrido, J. J. *et al.* A targeting motif involved in sodium channel clustering at the axonal initial segment. *Science* **300**, 2091, doi:10.1126/science.1085167 (2003).

37 Berkovic, S. F. *et al.* Benign familial neonatal-infantile seizures: Characterization of a new sodium channelopathy. *Ann Neurol* **55**, 550-557, doi:10.1002/ana.20029 (2004).

38 Ogiwara, I. *et al. De novo* mutations of voltage-gated sodium channel αII gene *SCN2A* in intractable epilepsies. *Neurology* **73**, 1046, doi:10.1212/WNL.0b013e3181b9cebc (2009).

39 Tavassoli, T. *et al. De novo SCN2A* splice site mutation in a boy with Autism spectrum disorder. *BMC Med Genet* **15**, 35, doi:10.1186/1471-2350-15-35 (2014).

40 Kwon, S. E. & Chapman, E. R. Synaptophysin regulates the kinetics of synaptic vesicle endocytosis in central neurons. *Neuron* **70**, 847-854, doi:10.1016/j.neuron.2011.04.001 (2011).

41 Tarpey, P. S. *et al.* A systematic, large-scale resequencing screen of X-chromosome coding exons in mental retardation. *Nat Genet* **41**, 535-543, doi:10.1038/ng.367 (2009).

42 Castle, C. D., Cassimere, E. K., Lee, J. & Denicourt, C. Las1L is a nucleolar protein required for cell proliferation and ribosome biogenesis. *Mol Cell Biol* **30**, 4404-4414, doi:10.1128/MCB.00358-10 (2010).

43 Castle, C. D., Cassimere, E. K. & Denicourt, C. LAS1L interacts with the mammalian Rix1 complex to regulate ribosome biogenesis. *Mol Biol Cell* **23**, 716-728, doi:10.1091/mbc.E11-06-0530 (2012).

44 Hu, H. *et al.* X-exome sequencing of 405 unresolved families identifies seven novel intellectual disability genes. *Mol Psychiatry* **21**, 133, doi:10.1038/mp.2014.193 (2015).
45 Wilson, M., Mulley, J., Gedeon, A., Robinson, H. & Turner, G. New X-linked syndrome of mental retardation, gynecomastia, and obesity is linked to DXS255. *Am J Med Genet* **40**, 406-413, doi:10.1002/ajmg.1320400405 (1991).
46 Singla, V., Romaguera-Ros, M., Garcia-Verdugo, J. M. & Reiter, J. F. *Ofd1*, a human disease gene, regulates the length and distal structure of centrioles. *Dev Cell* **18**, 410-424, doi:10.1016/j.devcel.2009.12.022 (2010).
47 Wang, J. *et al.* OFD1, as a ciliary protein, exhibits neuroprotective function in photoreceptor degeneration models. *PLoS One* **11**, e0155860, doi:10.1371/journal.pone.0155860 (2016).
48 D'Angelo, A. *et al.* OFD1 controls dorso-ventral patterning and axoneme elongation during embryonic brain development. *PLoS One* **7**, e52937, doi:10.1371/journal.pone.0052937 (2012).
49 Li, J. *et al.* Targeted sequencing and functional analysis reveal brain-size-related genes and their networks in autism spectrum disorders. *Mol Psychiatry* **22**, 1282-1290, doi:10.1038/mp.2017.140 (2017).
50 Cremers, F. P. *et al.* Deletions in patients with classical choroideremia vary in size from 45 kb to several megabases. *Am J Hum Genet* **47**, 622-628 (1990).
51 Li, S. *et al.* Exome sequencing reveals *CHM* mutations in six families with atypical choroideremia initially diagnosed as retinitis pigmentosa. *Int J Mol Med* **34**, 573-577, doi:10.3892/ijmm.2014.1797 (2014).
52 Lin, R. Y., Vera, J. C., Chaganti, R. S. & Golde, D. W. Human monocarboxylate transporter 2 (MCT2) is a high affinity pyruvate transporter. *J Biol Chem* **273**, 28959-28965 (1998).
53 Wiśniowiecka-Kowalnik, B. *et al.* Application of custom-designed oligonucleotide array CGH in 145 patients with autistic spectrum disorders. *Eur J Hum Genet* **21**, 620-625, doi:10.1038/ejhg.2012.219 (2013).
54 Yaron, A. & Zheng, B. Navigating their way to the clinic: emerging roles for axon guidance molecules in neurological disorders and injury. *Developmental Neurobiology* **67**, 1216-1231, doi:10.1002/dneu.20512 (2007).
55 Krumm, N. *et al.* Excess of rare, inherited truncating mutations in autism. *Nat Genet* **47**, 582-588, doi:10.1038/ng.3303 (2015).

Supplementary Table S5. Gene set enrichment analysis

Gene Set Category	# Genes in Gene Set (K)	# Genes in Overlap (k)	k/K	p-value	FDR q-value	Genes overlapped
GO Growth	990	6	0.0061	2.96E-06	1.50E-02	*ENPP1, IGF1, AGTR2, PLXNA3, MBD5, ATRX*
GO Developmental Growth	663	5	0.0075	8.22E-06	1.50E-02	*IGF1, AGTR2, MBD5, ATRX, PLXNA3*
GO Regulation of Transport	1842	7	0.0038	7.79E-06	1.50E-02	*ENPP1, IGF1, AGTR2, GRIN2B, SCN2A, DMD, SYP*
GO Homeostatic Process	1913	6	0.0031	1.23E-04	3.56E-02	*ENPP1, AGTR2, MBD5, ATRX, GRIN2B, DMD*
GO Head Development	771	5	0.0065	1.70E-05	1.89E-02	*AGTR2, PLXNA3, ATRX, GRIN2B, CHD8*
GO Synaptic Membrane	429	4	0.0093	3.37E-05	2.80E-02	*GRIN2B, SCN2A, DMD, SYP*
GO Central Nervous System Development	980	5	0.0051	5.36E-05	2.80E-02	*AGTR2, PLXNA3, ATRX, GRIN2B, CHD8*
GO Negative Regulation of Response to Stimulus	1655	6	0.0036	5.48E-05	2.80E-02	*ENPP1, IGF1, AGTR2, PLXNA3, DYRK1A, CHD8*
GO Iron Transport	1677	6	0.0036	5.90E-05	2.80E-02	*ENPP1, AGTR2, GRIN2B, SCN2A, DMD, SLC16A7*
GO Behavior	594	4	0.0067	1.19E-04	3.56E-02	*AGTR2, MBD5, GRIN2B, CHD8*

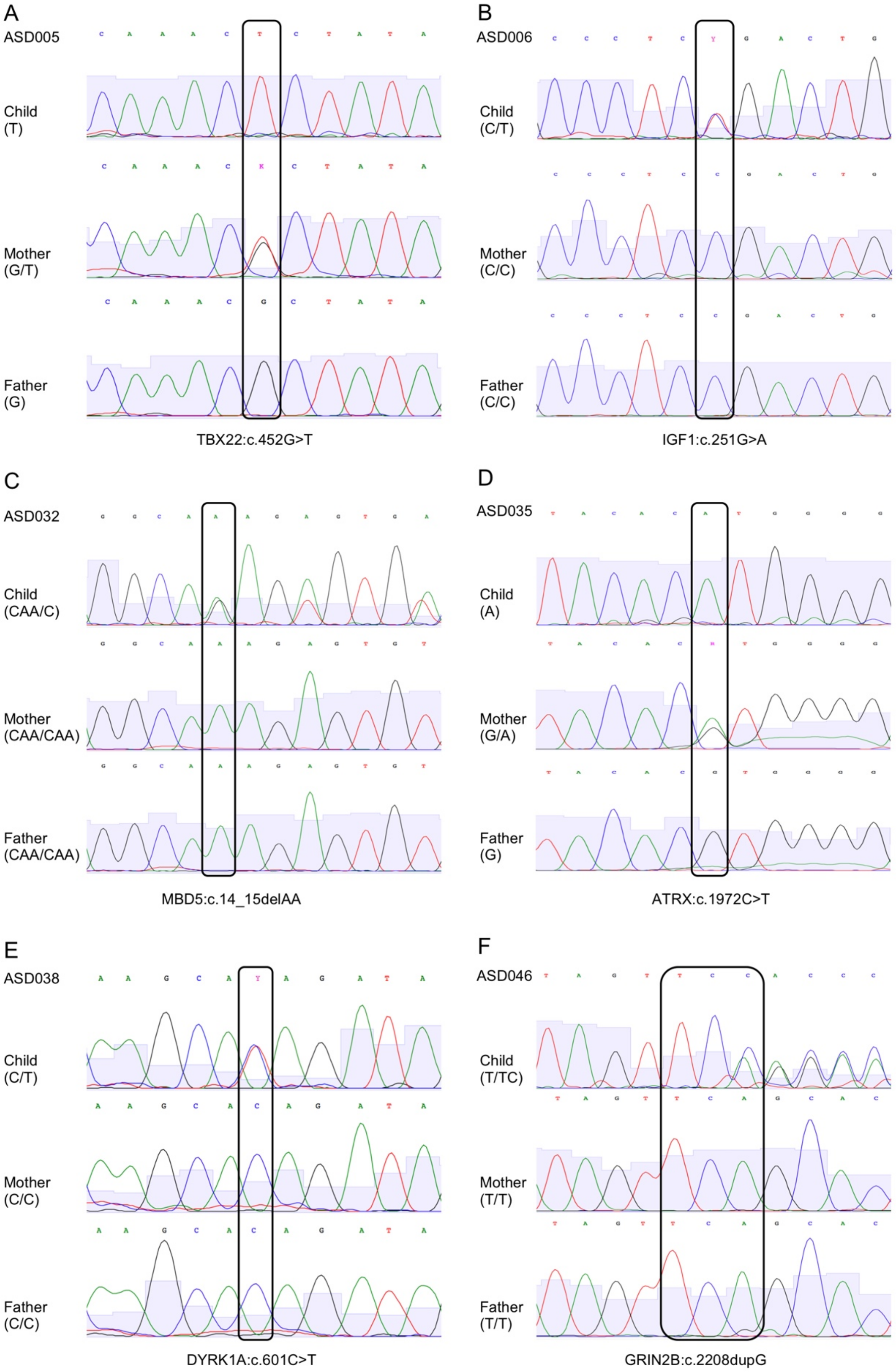
A
ASD005
Child
(T)
Mother
(G/T)
Father
(G)
TBX22:c.452G>T
B
ASD006
Child
(C/T)
Mother
(C/C)
Father
(C/C)
IGF1:c.251G>A
C
ASD032
Child
(CAA/C)
Mother
(CAA/CAA)
Father
(CAA/CAA)
MBD5:c.14_15delAA
D
ASD035
Child
(A)
Mother
(G/A)
Father
(G)
ATRX:c.1972C>T
E
ASD038
Child
(C/T)
Mother
(C/C)
Father
(C/C)
DYRK1A:c.601C>T
F
ASD046
Child
(T/TC)
Mother
(T/T)
Father
(T/T)
GRIN2B:c.2208dupG

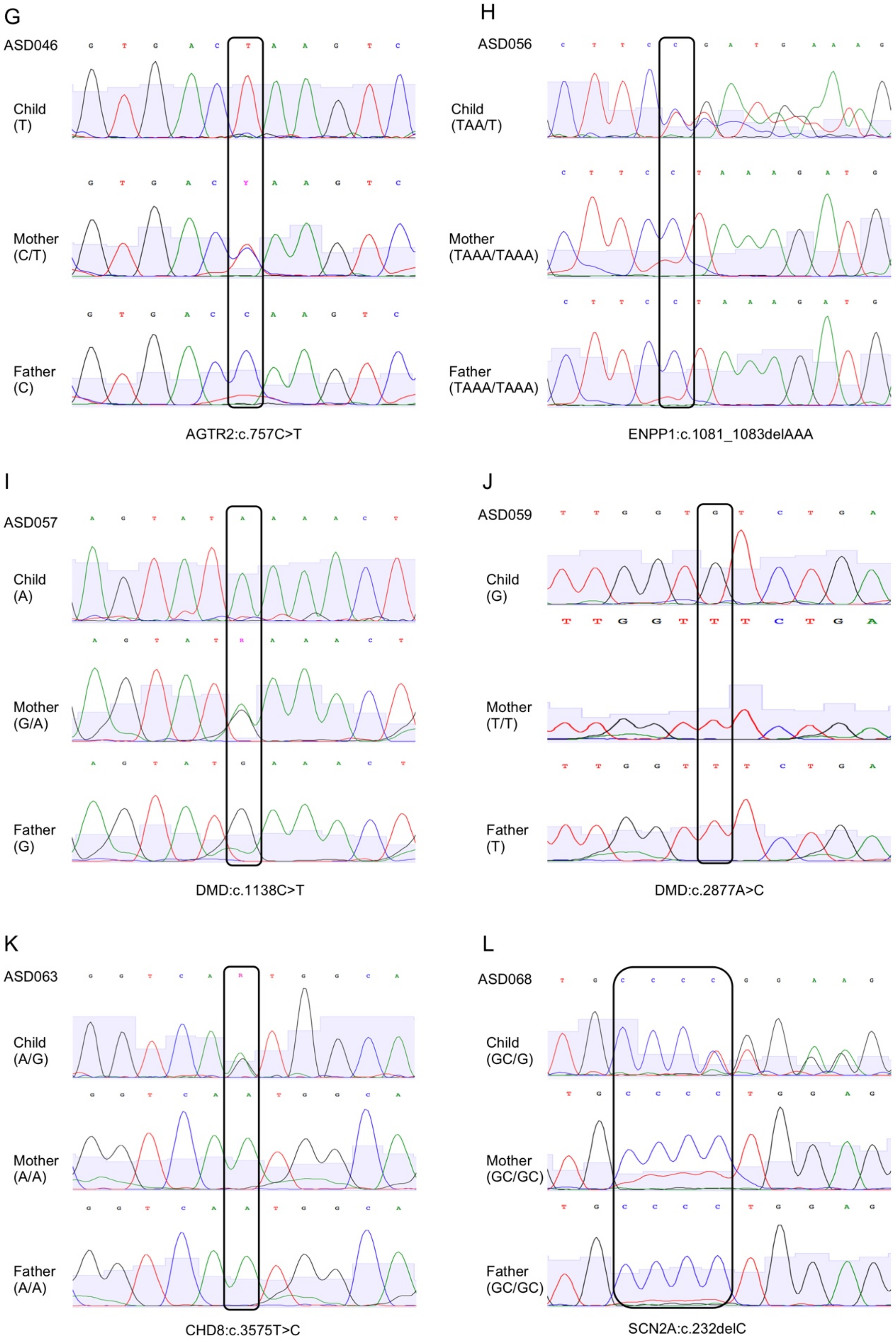
G
ASD046
Child
(T)
Mother
(C/T)
Father
(C)
AGTR2:c.757C>T
H
ASD056
Child
(TAA/T)
Mother
(TAAA/TAAA)
Father
(TAAA/TAAA)
ENPP1:c.1081_1083delAAA
I
ASD057
Child
(A)
Mother
(G/A)
Father
(G)
DMD:c.1138C>T
J
ASD059
Child
(G)
Mother
(T/T)
Father
(T)
DMD:c.2877A>C
K
ASD063
Child
(A/G)
Mother
(A/A)
Father
(A/A)
CHD8:c.3575T>C
L
ASD068
Child
(GC/G)
Mother
(GC/GC)
Father
(GC/GC)
SCN2A:c.232delC

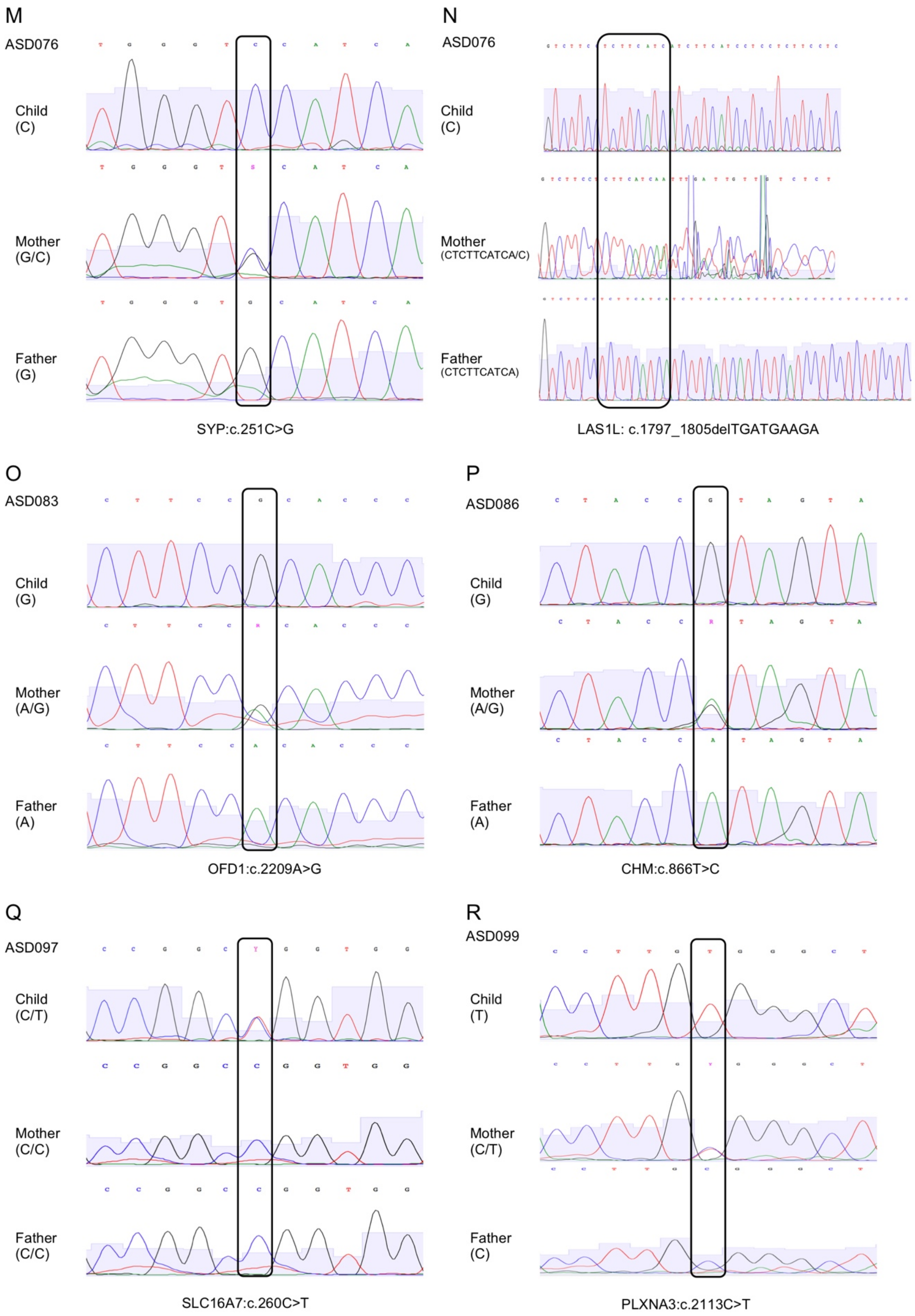

Supplementary Figure S1. Sanger validations of detected variants from the trios. (**A**) Trio ASD005. (**B**) Trio ASD006. (**C**) Trio ASD032. (**D**) Trio ASD035. (**E**) Trio ASD038. (**F, G**)

Trio ASD046. (**H**) Trio ASD056. (**I**) Trio ASD057. (**J**) Trio ASD059. (**K**) Trio ASD063. (**L**) Trio ASD068. (**M, N**) Trio ASD076. (**O**) P Trio ASD083. (**P**) Trio ASD086. (**Q**) Trio ASD097. (**R**) Trio ASD099. The horizontal line represents the trios with their genotypes in parentheses; The rectangle indicates the location of genetic predisposition in each proband compared to their parents.
